# The Use of Circulating Tumor DNA for Prognosis of Gastrointestinal Cancers

**DOI:** 10.3389/fonc.2018.00275

**Published:** 2018-07-24

**Authors:** Hariti Saluja, Christos S. Karapetis, Susanne K. Pedersen, Graeme P. Young, Erin L. Symonds

**Affiliations:** ^1^Flinders Centre for Innovation in Cancer, College of Medicine and Public Health, Flinders University, Bedford Park, SA, Australia; ^2^Department of Medicine, College of Medicine and Public Health, Flinders University, Bedford Park, SA, Australia; ^3^Department of Oncology, Flinders Medical Centre, Bedford Park, SA, Australia; ^4^Clinical Genomics Pty Ltd, North Ryde, NSW, Australia; ^5^Bowel Health Service, Flinders Medical Centre, Bedford Park, SA, Australia

**Keywords:** circulating tumor DNA, colorectal cancer, oesophageal cancer, gastric cancer, gastrointestinal stromal tumor, cell free circulating DNA, survival, recurrence

## Abstract

Gastrointestinal cancers, including oesophageal, gastric and colorectal cancers (CRC) have high rates of disease recurrence despite curative resection. There are a number of recent studies that have investigated the use of circulating tumor DNA (ctDNA) for prognostic value in these cancers. We reviewed studies that had been published prior to March 2018 that assessed the prognostic values of ctDNA in patients with oesophageal and gastric cancers, gastrointestinal stromal tumors (GIST) and CRC. We identified 63 eligible clinical studies that focussed on recurrence and survival. Studies assessed investigated various ctDNA biomarkers in patients with different stages of cancer undergoing surgical resection, chemotherapy and no treatment. For oesophageal squamous cell carcinoma and oesophageal adenocarcinoma, methylation of certain genes such as *APC* and *DAPK* have been highlighted as promising biomarkers for prognostication, but these studies are limited and more comprehensive research is needed. Studies focusing on gastric cancer patients showed that methylation of ctDNA in *SOX17* and *APC* were independently associated with poor survival. Two studies demonstrated an association between ctDNA and recurrence and survival in GIST patients, but more studies are needed for this type of gastrointestinal cancer. A large proportion of the literature was on CRC which identified both somatic mutations and DNA methylation biomarkers to determine prognosis. ctDNA biomarkers that identified somatic mutations were more effective if they were personalized based on mutations found in the primary tumor tissue, but ctDNA methylation studies identified various biomarkers that predicted increased risk of recurrence, poor disease free survival and overall survival. While the use of non-invasive ctDNA biomarkers for prognosis is promising, larger studies are needed to validate the clinical utility for optimizing treatment and surveillance strategies to reduce mortality from gastrointestinal cancers.

## Introduction

Gastrointestinal cancers, in particular gastric (stomach) and colorectal cancer (CRC), have high incidence and mortality rate. CRC is one of the most prevalent cancers, with ~1.4 million new cases diagnosed and 693,933 deaths worldwide per year in 2012 (1). The incidence and mortality rate of gastric cancer during the same period was 951,594 and 723,073 respectively, while oesophageal (adenocarcinoma or squamous cell carcinoma) cancer incidence was 455,784 with a mortality of 400,169. From this it can be seen that while the incidence of oesophageal cancer is less common than gastric cancer and CRC, it has a higher mortality rate which is due to the tumors being rarely detected before the disease has metastasized to lymph nodes and distant organs (2, 3). Gastrointestinal stromal tumors (GIST) are mesenchymal tumors that can originate anywhere in the gastrointestinal tract, but with a higher proportion in the stomach and small intestine. They are not common and are thought to make up <1% of all gastrointestinal tumors. Five year survival from this cancer is ~50% (2, 4).

In recent years there have been large improvements in early detection, surgical resection and treatment of gastrointestinal cancers, especially colorectal and gastric. Despite this, the risk of recurrence of the cancer within 5 years is reported to be up to 50% (4–6). For oesophageal cancer and GIST approximately half of the patients undergoing curative resection develop recurrence (4, 5), with half of the oesophageal recurrences occurring within the first year post-resection (5). Despite deeming patients free of disease at the conclusion of initial therapy, gastric cancer has been reported to have a recurrence incidence of 26% (7), and following CRC resection, incidence of recurrence is ~35%, with 80% occurring within the first 2 years of resection (6). Metastatic recurrence is most commonly detected too late for successful intervention (8, 9) although for CRC at least early detection of tumor progression and recurrence provides an additional effective way to improve clinical outcomes. Accurate prognostic assessment to allow for early and effective treatment is vital to improving patient outcomes.

## Current tools for prognosis and surveillance

Assessment of prognosis and determining treatment and surveillance strategies is currently guided by the stage at diagnosis which is classified according to the T, N, M system, i.e., depth of tumor invasion (T stage), presence of lymph node metastasis (N stage), and presence of distance metastasis (M stage) (10). Staging of GIST is based on tumor size instead of depth of invasion, with mitotic rate combined with T, N, and M scores to give an overall stage (11). While the use of TNM stage is highly prognostic for stage I and IV, it is less predictive for stage II and III. For example, patients with stage II CRC are considered to have low risk for recurrence and therefore are not generally recommended adjuvant chemotherapy, but despite this, one study found that 23% of stage II patients had a recurrence within 5 years (12). Consequently, other clinicopathological factors have been sought to help predict who is at greatest risk for recurrence (examples provided in Table 1). For oesophageal cancer increased depth of tumor invasion correlates with an increased risk of disease recurrence (14), however it is not highly predictive of which patients are at low or high risk for disease recurrence. Similarly for CRC, T stage, vascular invasion, tumor grade, and number of examined lymph nodes have been associated with poor prognosis (Table 1), however, recurrence still occurs in patients without these risk factors (31).

**Table 1:** Significant predictive clinicopathological factors of recurrence for gastrointestinal cancers on multivariate analysis.

**Patient group**	**Clinicopathological variable**	**Multivariate analysis findings**
Oesophageal cancers (73.3% adenocarcinomas) (13)	Poor differentiation	HR 1.74; 95%CI 1.28–2.38
	Advanced clinical stage	HR 6.46; 95%CI 2.90–14.38
Oesophageal cancers (82.5% squamous cell carcinoma) (14)	Depth of tumor invasion	RR 1.9; 95%CI 1.3–2.7
Gastric cancer (15)	Age at diagnosis	OR 1.813; 95%CI 1.050–3.131
	T stage	OR 2.865; 95%CI 1.603–5.123
	N1 stage (vs. N0)	OR 4.029; 95%CI 1.708–9.500
	N2 stage (vs. N0)	OR 4.425; 95%CI 1.889–10.365
	N3 stage (vs. N0)	OR 9.860; 95%CI 4.314–22.536
	Lauren histotype	OR 3.492; 95%CI 1.810–6.736
	Lymphovascular invasion	OR 3.460; 95%CI 1.335–8.969
Gastric cancer (lymph node negative) (16)	T stage ≥3	SHR 2.7; 95%CI 1.5–5.2
Gastric cancer (lymph node negative) (17)	Diffuse + mixed histotype (vs. intestinal)	RR 2.11; 95%CI 1.25–2.95
	T3 stage (vs. T2)	RR 3.55; 95%CI 1.98–6.44
Gastrointestinal stromal tumor (18)	Mitotic index 6-10/50 HPF (vs. ≤5/50)	RR 0.282; 95%CI 0.121–0.660
	Platelet to lymphocyte ratio	RR 1.737; 95%CI 1.041–2.899
	Gastrointestinal bleeding	RR 0.457; 95%CI 0.254–0.823
Gastrointestinal stromal tumor (19)	High risk	HR 13.01; 95%CI 2.68–63.21
	Omental/colorectal site	HR 5.13; 95%CI 1.68–15.69
	Age at diagnosis	HR 0.96; 95%CI 0.92–0.99
Gastrointestinal stromal tumor (20)	Size ≥5 cm (vs. <5 cm)	HR 3.43; 95%CI 1.12–11.8
	Mitotic index ≥5 (vs. <5/50 HPF)	HR 3.28; 95%CI 1.25–8.59
Gastrointestinal stromal tumor (21)	Female	HR 0.469; 95%CI 0.257–0.854
	Size ≥10 cm (vs. <5 cm)	HR 20.989; 95%CI 3.560–125.673
	Epithelioid component	HR 5.315; 95%CI 1.402–20.149
	Mitotic index ≥10 (vs. <10)	HR 45.951; 95%CI 8.811–239.657
Gastrointestinal stromal tumor (22)	Size ≥10 cm (vs. <10 cm)	OR 4.715; 95%CI 1.142–19.471
Colon cancer (stage I-III) (23)	Stage II (vs. I)	HR 4.6; 95%CI 1.05–19.9
	Stage III (vs. I)	HR 10.8; 95%CI 2.6–45.8
	Clinical obstruction	HR 3.8; 95%CI 1.9–7.4
	Positive margin	HR 4.1; 95%CI 1.9–8.6
	Lymphovascular invasion	HR 1.9; 95%CI 1.06–3.5
	Local tumor invasion	HR 2.2; 95%CI 1.1–4.5
Colon cancer (stage III) (24)	Positive lymph node	HR 1.24; 95%CI 1.18–1.31
Colon cancer (stage I-III) (25)	4.0–7.9 cm (vs. <4 cm)	HR 0.45; 95%CI 0.293–0.696
	Venous invasion	HR 1.61; 95%CI 1.085–2.376
	Stage III (vs. stage I)	HR 3.80; 95%CI 1.482–9.744
Rectal cancer (stage I-III) (25)	Lower rectum (vs. rectosigmoid)	HR 2.20; 95%CI 1.408–3.424
	Anal canal (vs. rectosigmoid)	HR 7.19; 95%CI 3.052–16.950
	Serosal invasion	HR 1.63; 95%CI 1.130–2.343
	Venous invasion	HR 1.90; 95%CI 1.407–2.566
	Stage III (vs. stage I)	HR 3.64; 95%CI 1.993–6.634
	Questionable residual tumor	HR 1.84; 95%CI 1.281–2.634
Rectal cancer (stage III) (26)	tumor budding	HR 2.005; 95%CI 1.021–3.934
	N stage	HR 1.818; 95%CI 1.057–3.128
	Perineural invasion	HR 1.046; 95%CI 1.011–1.081
	T stage	HR 1.606; 95%CI 1.149–2.244
Colorectal cancer (stage I-III) (27)	Vascular invasion	HR 2.304; 95%CI 1.067–4.975
	Perineural invasion	HR 3.040; 95%CI 1.389–6.667
Colorectal cancer (stage I-III) (28)	Lymph node metastases	HR 7.652; 95%CI 4.162–14.827
	Vascular invasion	HR 4.360; 95%CI 2.793–10.847
Colorectal cancer (stage II) (29)	T4 stage (vs. T3)	HR 23.072; 95%CI 2.951–203.247
	Vascular invasion	HR 6.204; 95%CI 2.879–12.694
	≥12 lymph nodes retrieved	HR 2.656; 95%CI 1.319–6.127
Colorectal cancer (stage IV) (30)	High grade differentiation	HR 1.514; 95%CI 1.124–2.040
	Curative operation	HR 2.642; 95%CI 1.966–3.549
	Resection of primary tumor	HR 0.507; 95%CI 0.366–0.704
	Multiple metastatic lesions	HR 1.679; 95%CI 1.165–2.418

*95% CI, confidence interval; HR, hazard ratio; OR, odds ratio; SHR, subhazard ratio*.

Intensive surveillance after resection may be applied to detect asymptomatic recurrence early enough to enable curative therapy and improve survival. Current methods for surveillance post-resection are radiological techniques (e.g., CT) and endoscopy with biopsy, but these have disadvantages of radiation burden, lack of sensitivity, invasive nature, and cost as well as limited resources in some countries. Biopsy only samples a small area of the epithelium which might not be representative of the stage of disease (3). In addition the cost effectiveness of intensive surveillance has been questioned (32). Oesophageal cancers may also benefit from surveillance with cytological sampling obtained by brushing the oesophageal surface, or use of the capsule sponge, but these techniques are also limited by inadequate sensitivity and specificity, or limited validation (3). There remains a need for non-invasive and sensitive prognostic markers to establish who would benefit from adjuvant therapy and surveillance. This could be done through pre- or post-operative blood analysis.

## Assessment of blood for biomarkers of cancer prognosis

The use of blood biomarkers has the potential to provide further prognostic information of value for gastrointestinal cancers, however, current clinical use is limited. Blood testing for proteins are not routinely used for oesophageal cancers or GIST, but the proteins carcinoembryonic antigen (CEA), carbohydrate antigen-19-9 (CA 19-9), and carbohydrate antigen 72-4 (CA 72-4) have been used for monitoring disease progression of CRC and gastric cancer (33). For CEA, while it is upregulated in 90% of advanced CRC (34), it is not reliably used for prognosis, and studies have shown an unacceptably low sensitivity for recurrent CRC of 32–37% (35–37). The sensitivity of CEA for recurrence in gastric cancer has been reported to be between 30.8 and 34.3% (38, 39). There is a wide range of sensitivity of CA 19-9 of 30.8–57.1% (38, 39), with a similar average sensitivity of CA 72-4 of 48.4% (39). The low sensitivity supports the need for other blood biomarkers for clinical management to assess risk for recurrence.

Genetic markers arising from tumors and being released into blood might provide the solution. Most gastrointestinal cancers are thought to develop through a series of epigenetic changes or somatic (non-hereditary) lesions. The common mutations are in genes including *APC, TP53, KRAS* and *BRAF* for colorectal cancer [reviewed in Testa et al. (40)], *TP53* and *p16/CDKN2A* in oesophageal adenocarcinomas [reviewed in Testa et al. (41)]*, CDH1, PIK3CA*, and *RHOA* in gastric cancers [reviewed in Ang et al. (42)], and *KIT* and *PDGFRA* with GIST [reviewed in Wozniak et al. (43)]. These alterations can contribute to aberrant cell behavior such as uncontrolled cell growth and proliferation, disordered apoptosis, increased angiogenesis, and promotion of invasion and metastasis (44). As primary and metastatic cancers shed cells, cell components and DNA into the blood, these genetic changes can be monitored in the circulation by assaying for circulating tumor cells (CTCs) or for circulating tumor DNA (ctDNA), with the process sometimes referred to as liquid biopsy. While CTCs show promise in early detection of recurrence [reviewed in Tan et al. (45)], clinical use is limited by low numbers of CTCs in the blood (one mL of whole blood generally contains less than one CTC, but 10^7^ normal blood cells) (46). Furthermore, CTCs show heterogeneity such that extraction techniques might not be effective for all cell types; CTCs can be comprised of epithelial tumor cells, epithelial-to-mesenchymal transition (EMT) cells, and stem cells (46). The use of ctDNA which is more abundant and easier to extract, overcomes some of the technical issues associated with using CTCs in clinical practice and captures the genetic material released independent of cell structure. ctDNA remains in the circulation for a few hours before being metabolized, (47) which allows real-time monitoring of the tumor burden, with a comprehensive molecular profile of the heterogeneity of the disease, compared to what is provided by a single tumor tissue biopsy (48).

The release of ctDNA into the bloodstream as cell free DNA (cfDNA) is thought to be the result of apoptosis or necrosis of tumor cells (49). When DNA is released through necrosis of cells, the fragments can vary in size, whereas DNA released through apoptosis creates fragments 185–200 base pairs in length (50). As the main source of DNA from non-neoplastic healthy cells is apoptosis, assessment of the ratio of longer DNA to short fragments (through measuring ALU repeats) is able to indicate presence of ctDNA (51). Other common strategies involve assessing cell free DNA levels, tumor specific DNA mutations, and tumor specific epigenetic changes. The latter two can be assessed through targeted PCR-based ctDNA assays, detecting known somatic mutations or epigenetic changes. One such example is assessment of *RAS* mutations of colorectal cancer tissue which are of similar prevalence in plasma as in the tumor (51 and 53% respectively), demonstrating that blood-based testing for RAS mutation is a viable alternative to tissue-based testing (52). A growing number of studies have assessed DNA methylation as there is evidence that epigenetic alterations are more common and frequently precede mutational (somatic) changes (53). Also unlike mutations, promoter methylation can be consistently measured as it occurs in specific regions of the DNA (CpG islands).

CtDNA has been evaluated as a screening tool and for diagnostic purposes, but there has been limited effectiveness with early stage cancers and it does not appear useful in predicting the presence of colonic polyps (54). Instead the use of ctDNA for prognosis and treatment monitoring is more promising. The following sections of this review will describe the studies that have been performed in gastrointestinal cancers to assess the utility of ctDNA for their prognostic value, whether measured as cfDNA concentration, integrity (fragment lengths), copy number alterations, mutation or methylation status. These are comprehensively summarized in Supplementary Tables 1–4.

## Search strategy

Identification of eligible studies was performed through searching the PubMed database until 1st March 2018. The following search criteria were applied: “(ctDNA OR “circulating tumor DNA” OR “tumor derived DNA” OR “circulating tumor DNA” OR “tumor derived DNA” OR “cell free DNA”) AND (gastrointestinal OR GIT OR esophagus OR esophagus OR oesophageal OR esophageal OR gastroesophageal OR stomach OR gastric OR “large intestine” OR colon OR caecum OR rectum OR colorectal) AND (tumor OR tumor OR malignan^*^ OR cancer OR neoplasm OR carcinoma OR carcinoid OR adenocarcinoma).” This resulted in 657 search results. Two independent reviewers (HS and ES) screened the available literature, and discrepancies were discussed and resolved. Included studies were those conducted in gastrointestinal cancers with a clinical outcome of survival or recurrence. Exclusions were review articles, biomarker studies that did not include blood analysis, studies in animal models or cell lines only, articles that were not in English, and those that analyzed circulating tumor cells (CTC) rather than circulating tumor DNA or cell free DNA. In the case of more than one report on the same cohort of patients, the study with the shorter follow-up time was excluded. In addition, studies were not included where the focus was on associations of biomarkers with pathology indicators of poor prognosis, rather than an actual clinical outcome of poor prognosis. The final number of eligible studies for review were 63, including 7 on oesophageal cancers, 13 on gastric cancers, 2 on GIST, and 41 on CRC.

## ctDNA biomarkers for prognosis of oesophageal cancer

Biomarkers for prognosis have been investigated for both adenocarcinomas and squamous cell carcinomas of the esophagus as summarized below.

### DNA levels, integrity, and copy numbers

It was previously shown that cfDNA levels correlated with stage in oesophageal squamous cell carcinoma (SCC). Tomochika et al (*n* = 91) found that DNA levels were higher in advanced tumors vs. early stages, and significantly higher in patients with distant metastases (*p* = 0.011) (55). Correlation of DNA levels before oesophagectomy for stage I-III SCC were also observed with tumor lymphovascular invasion and relapse (*p* = 0.018), and a poor 5 year disease free survival rate in 81 oesophageal SCC patients (*p* = 0.013) (56).

### DNA mutations

Ueda et al conducted a longitudinal study to look at 53 cancer related genes in 13 oesophageal SCC patients undergoing surgery of all stages. Changes in allele frequency in ctDNA was associated with tumor burden, and the allelic frequency increased prior to radiographic detection of recurrence (6 months before radiological evidence) (57). Eisenberger et al assessed loss of heterozygosity (LOH) in pre-operative ctDNA of SCC (*n* = 28) and oesophageal adenocarcinoma (*n* = 32) patients of all stages in two separate studies. In both types of cancers, no relationship was found between recurrence and LOH; however, in SCC a trend toward shorter survival was observed for patients with LOH in tumor tissue and ctDNA (58, 59).

### DNA methylation

Of the few studies that have assessed prognostic value of methylated ctDNA biomarkers in oespophageal cancer, there have been mixed outcomes, which may be related to different cancer types studied. Presence of high pre-operative methylated ctDNA (*MSH2*) was predictive of lower disease free survival for 209 SCC patients of all stages (60), while in all stages of oesophageal adenocarcinomas pre-operative methylated ctDNA (*TAC1*) was not associated with survival (*n* = 61) (61). Hoffman et al assessed methylation of *DAPK* and *APC* promoter in 24 SCC and 35 adenocarcinoma patients of stage 0-III at pre- and post-operative stages. Presence of pre-operative *DAPK* methylation was associated with poorer survival (*p* = 0.01) and detection of post-operative methylation of *APC* promoter was correlated with residual tumor (*p* = 0.03) (62).

### Summary

There have been a limited number of studies undertaken to develop prognostic biomarkers with oesophageal SCC and adenocarcinoma. Some of these studies are highlighted in Table 2 [limiting the studies displayed to those with at least 20 events of interest (recurrence or death)], but there have been very limited accuracy data for each test. Only a test utilizing copy numbers was assessed for sensitivity for recurrence (61.2%), but specificity was not assessed (56).

**Table 2:** Accuracy of circulating tumor biomarkers for oesophageal cancer recurrence and survival (excluding duplicate studies and those with unclear number or fewer than 20 recurrences or deaths).

**Author, year**	**Biomarkers**	**Stage**	**Pre-op, post-op or time of recurrence**	**No. recurrence/total**	**Sensitivity for recurrence**	**Specificity for recurrence**	**NPV for recurrence**	**PPV for recurrence**	**HR for recurrence (95% CI)**	**Adjusted HR (95% CI)**	**Mean/Median recurrence-free survival**
**RECURRENCE**											
Eisenberger; 2006 (59)	12 microsatellite markers that indicate LOH	Adenocarcinoma; all stages	Pre-op	22/32	N/A	N/A	N/A	N/A	N/A	N/A	No association with recurrence.
Hseih; 2016 (56)	DNA copy number (cyclophilin)	Squamous cell carcinoma; stage I-III	Post-op	49/81	61.2%	N/A	N/A	N/A	N/A	N/A	N/A
**SURVIVAL**											
**Author, year**	**Biomarkers**	**Stage**	**Pre-op or post-op**	**No. deaths/total**	**Sensitivity for death**	**Specificity for death**	**NPV for death**	**PPV for death**	**HR for survival (95% CI)**	**Adjusted HR (95% CI)**	**Mean/Median survival**
Eisenberger; 2006 (59)	12 microsatellite markers that indicate LOH	Adenocarcinoma; all stages	Pre-op	24/34	N/A	N/A	N/A	N/A	N/A	N/A	No association with survival.
Hseih; 2016 (56)	DNA copy number (cyclophilin)	Squamous cell carcinoma; stage I–III	Post-op	43/81	N/A	N/A	N/A	N/A	N/A	N/A	24.3% (vs. 43.4%), *p* = 0.164

*CI, Confidence interval; HR, hazard ratio; N/A, not applicable; NPV, negative predictive value; PPV, positive predictive value; post-op, post-operative; pre-op: pre-operative*.

## ctDNA biomarkers for prognosis of gastric cancer

### DNA levels, integrity and copy numbers

A number of studies have investigated the use of cfDNA levels to determine the clinical outcome following surgical resection of gastric cancer. Kim et al (*n* = 30) and Pu et al (*n* = 73) provided data that supported that advanced gastric cancer (stage III/IV) patients had higher levels of DNA compared with early gastric cancer patients (*p* = 0.035) (63, 64). Pu et al conducted a longitudinal study and found that DNA levels were elevated pre-operatively and at 21 days post-operatively; but they declined 3 months post-surgery and then increased again if the patient had tumor progression. However this study showed no significant association of DNA levels with survival (64). A large study of 428 gastric cancer patients by Lan et al found that persistently high DNA levels post-resection was an indicator of recurrence (65). In a study that focussed on 277 stage IV cases it was found that a high level of DNA with more mutations was present pre-operatively (*p* < 0.0001) and these patients had an increased risk of recurrence (*p* = 0.037) and lower overall survival (*p* = 0.039) over the 5-year follow-up period (8). Several studies have also assessed DNA copy numbers for prognostic purposes. A study by Shoda et al examined 61 stage I and II surgical resection patients and found that *HER2* to *RPPH1* ratio of ctDNA increased post-operatively with recurrence (66). In a separate study, this research group looked at the value of EBV (Epstein–Barr virus) DNA in 153 gastric cancer patients undergoing resection. In the 21 (13.7%) patients with EBV-associated gastric carcinoma, circulating EBV DNA levels reflected the clinical status of the patient as it was absent after surgery in all 9 cases assessed, and increased prior to clinical detection of recurrence in one patient with longitudinal follow-up over 2 years (67). While plasma EBV DNA may useful for monitoring clinical load in patients with EBV-associated gastric carcinomas, no significant difference was found between prognosis of recurrence-free survival of those with high pre-operative EBV copy numbers compared to those with low levels (67). Kinugasa et al (68) assessed the ctDNA *HER2* status in relation to survival of patients with non-resectable gastric cancer (2 stage III and 23 stage IV). They reported that patients with a positive pre-therapy *HER2* ctDNA status had significantly shorter survival than patients with a negative status (*p* = 0.01). However, as a poor concordance was found between tissue and serum *HER2* status, only 3 of the 7 patients that were ctDNA *HER2* positive were also positive with tissue biopsy and received directed therapy (trastuzumab). No difference in survival was found when comparing survival rates of patients with a positive or negative *HER2* status of the tissue. Caution must therefore be taken in interpreting the prognostic value of *HER2* ctDNA status.

### DNA mutations

Very few studies have assessed DNA mutations for gastric cancer prognosis. One of the studies was a longitudinal study in 42 stage II gastric cancer patients undergoing surgical resection which evaluated concentration of *TP53* mutations. It was found that the change in ctDNA fraction corresponded with disease status of the patients i.e. the levels decreased post-operatively but increased in patients with recurrence (69). However, the authors did not perform statistical analyses on these results as there were only 3 cases with recurrence.

### DNA methylation

A few papers have studied methylation of ctDNA in gastric cancer and found a significantly worse clinical outcome in patients who have aberrant methylation of various genes in ctDNA. Pimson et al found 85 and 95% of 101 advanced gastric cancer patients had *PCDH10* and *RASSF1A* methylation which was associated with a reduction in median survival to ~8 months (*p* < 0.001) (70). Balgkouranidou et al also studied *RASSF1A* methylation, along with *APC* methylation, in 73 operable gastric cancer patients of stage I-III and did not find a significant correlation with *RASSF1A* promoter methylation and clinical outcome; but showed that the group with pre-operative *APC* promoter methylation had a higher incidence of death (HR 4.6, *p* = 0.008). *APC* methylation levels were also associated with high levels of the conventional tumor biomarkers, CEA and CA19-9 (71). In a similar study, Balgkouranidou et al found that methylation of *SOX17* in pre-operative ctDNA of 73 patients with operable gastric cancer had decreased overall survival (*p* = 0.049) (72). Two studies investigated different ctDNA biomarkers, *MINT2* promoter and *TIMP*-*3* respectively, for disease-free progression and risk of recurrence in the same population of 92 gastric cancer patients of all stages undergoing surgical resection. Aberrant methylation of *MINT2* promoter in pre-operative ctDNA was associated with peritoneal dissemination and tumor progression (*p* < 0.0001); and methylation of *TIMP*-*3* was associated with poorer disease free survival rates (*p* < 0.001) (73, 74). A study by Ling et al assessed *XAF1* methylation in pre-operative and post-operative follow-up ctDNA of 202 gastric cancer patients of all stages and showed that negative to positive methylation change post-surgery was associated with a poorer disease-free survival (*p* < 0.0001) (75).

### Summary

As with oesophageal cancers, there have been few thorough studies into ctDNA for prognosis of gastric cancer (Table 3) and none have shown to be an independent predictor for recurrence. Methylation changes appear to be the most promising with methylated *RASSF1A* and *SOX17* being independent predictors of overall survival. Despite this, the sensitivity and positive predictive value reported for some of these biomarkers may not be sufficiently high enough to guide therapeutic decisions.

**Table 3:** Accuracy of circulating tumor biomarkers for gastric cancer recurrence and survival (excluding duplicate studies and those with unclear number or fewer than 20 recurrences or deaths).

**Author, year**	**Biomarkers**	**Stage**	**Pre-op, post-op or time of recurrence**	**No. recurrence/total**	**Sensitivity for recurrence**	**Specificity for recurrence**	**NPV for recurrence**	**PPV for recurrence**	**HR for recurrence (95% CI)**	**Adjusted HR (95% CI)**	**Mean/Median recurrence-free survival**
**RECURRENCE**											
Fang; 2016 (8)	DNA copy number (cyclophilin)	All stages	Pre-op	129/244	49.6%	N/A	N/A	N/A	1.19 (0.86–1.63)	N/A	N/A
**SURVIVAL**											
**Author, year**	**Biomarkers**	**Stage**	**Pre-op or post-op**	**No. deaths/total**	**Sensitivity for death**	**Specificity for death**	**NPV for death**	**PPV for death**	**HR for poor survival (95% CI)**	**Adjusted HR (95% CI)**	**Mean/Median survival**
Fang; 2016 (8)	DNA copy number (cyclophilin);	All stages	Pre-op	179/277	N/A	N/A	N/A	N/A	N/A	N/A	High DNA levels: 39.2% (vs. 45.8%), *p* = 0.039
	DNA mutations										DNA mutations stage III-IV: 5.6% (vs. 31.5%), *p* = 0.028
Balgkouranidou; 2013 (72)	Methylation: *SOX17*	Operable gastric cancer	Pre-op	38/73	68%	51%	60%	61%	2.0 (1.0–3.9)	3.0 (1.2–7.8)	37.7 months (vs. 66.9 months), *p* = 0.049
Balgkouranidou; 2015 (71)	Methylation: *APC, RASSF1A*	Stage I-III	Pre-op	38/73	*APC:* 94.7% *RASSF1A:* 65.8%	N/A	N/A	*APC:* 59.0% *RASSF1A:* 56.5%	N/A	*APC:* 4.6 (1.1-20.3) *RASSF1A:* Not significant	*APC:* 46.0 months (vs. 85.0 months), *p* = 0.008 *RASSF1A:* 56.0 months (vs. 43.0 months), *p* = 0.683

*CI, Confidence interval; HR, hazard ratio; N/A, not applicable; NPV, negative predictive value; PPV, positive predictive value; post-op, post-operative; pre-op: pre-operative*.

## ctDNA biomarkers for prognosis of gastrointestinal stromal tumors

Two studies looked at the role of ctDNA in prognosis of GIST. In 92 patients with recurrent GIST, Rawnaq et al found an association between loss of heterozygosity in microsatellite DNA and recurrence (*p* = 0.03), but no association with overall survival (76). A study by Yoo et al on 30 patients with tyrosine kinase inhibitor-refractory GIST found that a detection of secondary kinase mutations (*KIT* exon 17) prior to treatment was associated with lower overall survival (HR 2.7, 0.047) (77).

### Summary

Only the study by Rawnaq et al. (76) had a moderate sample size, and has been summarized in Table 4. There have been no investigations into methylation markers of ctDNA, and the existing studies have not found a biomarker that is an independent predictor of either recurrence or survival. More studies are clearly needed for this type of gastrointestinal cancer.

**Table 4:** Accuracy of circulating tumor biomarkers for gastrointestinal stromal tumor recurrence (excluding duplicate studies and those with unclear number or fewer than 20 recurrences).

**Author, year**	**Biomarkers**	**Stage**	**Pre-op, post-op or time of recurrence**	**No. recurrence/total**	**Sensitivity for recurrence**	**Specificity for recurrence**	**NPV for recurrence**	**PPV for recurrence**	**HR for recurrence (95% CI)**	**Adjusted HR (95% CI)**	**Mean/Median recurrence-free survival**
**RECURRENCE**											
Rawnaq; 2011 (76)	12 microsatellite markers for loss of heterozygosity	Advanced or recurrent GIST	Post-op	29/83	58%	75%	N/A	N/A	N/A	N/A	N/A

*CI, Confidence interval; HR, hazard ratio; N/A, not applicable; NPV, negative predictive value; PPV, positive predictive value; post-op, post-operative; pre-op: pre-operative*.

## ctDNA biomarkers for prognosis of colorectal cancer

### DNA levels, integrity and copy numbers

As demonstrated with other gastrointestinal cancers, level of cfDNA correlates with presence and stage of tumors. Metastatic CRC was found to have highest cfDNA levels, with these decreasing for all patients post resection (*n* = 205) (51). Cassinotti et al and Frattini et al noted that DNA levels increased prior to recurrence in all stages of CRC (*n* = 223, *n* = 70) (78, 79). In two different studies of 38 primary CRC patients, Czeiger and colleagues found that pre-operative DNA level was a better indicator of prognosis than TNM staging for both disease-free survival (HR 6.03) and overall survival (HR 3.53) for all cancer stages. They also showed that DNA levels out-performed pre-operative CEA results, which was not significantly associated with disease-free survival (80, 81). Guadaljara et al found that a high level of pre-operative cfDNA in all CRC stages was correlated with presence of metastases at the time of the surgery or during follow up, but was not associated with overall survival (*n* = 73) (82). Schwarzenbach et al only assessed 55 stage IV CRC patients and found that high DNA levels prior to surgical resection was associated with a shorter survival period (83). Shorter overall survival has also been found to be associated with high pre-operative DNA levels measured as DNA fragments (ALU244 and ALU83, which are thought to represent the amount of the DNA released from non-apoptotic process and the total cfDNA) and DNA copy numbers (measured with DNA binding protein CPP1; *n* = 114, *n* = 45 respectively) (84, 85).

In metastatic CRC patients being treated with chemotherapy, high levels of cfDNA correlated with a worse outcome for the patient. Spindler et al (*n* = 100) found patients with high level of DNA prior to second-line treatment with irinotecan had shorter progression-free survival and overall survival (*p* < 0.0001) (86). In another study Spindler et al assessed 229 patients with chemorefractory metastatic CRC, and patients with high DNA levels had an impaired overall survival, with each increase in cfDNA quartile having an independent prognostic value (*p* = 0.0006) (87). In 49 patients with therapy resistant metastatic CRC being treated with gemcitabine and capecitabine, it was shown that high DNA levels prior to therapy was associated with lower overall survival (88). Schou et al assessed cfDNA levels longitudinally in 123 patients with locally advanced rectal cancer receiving chemotherapy and found that a high baseline level was associated with a higher risk of local/distant recurrence and a shorter time to recurrence (*p* = 0.002) (89).

### DNA mutations

The relationship between pre-operative ctDNA and survival or recurrence, using mutation markers, in CRC patients of all stages has been explored in a number of studies. Lin et al quantified ctDNA by amplifying mutations in 74 genes and showed that ctDNA, lower than the median value, was associated with a higher 5-year overall survival (*p* = 0.001) (*n* = 191) (90). Möhrmann et al assessed mutations in *BRAF, KRAS*, and *EGFR* genes in ctDNA of 20 advanced CRC patients and also found that lower ctDNA corresponded with longer survival (91). These findings were supported by a study of 37 patients that evaluated the presence of *KRAS* mutations and p16 hypermethylation in all stages of CRC and found a strong association between detection of ctDNA, and a shorter survival and higher risk of recurrence (92). Similarly, Wang et al concluded that detection of genetic alterations in *APC, p53*, and *KRAS* in a sample of 104 pre-operative CRC patients was linked to increased incidence of recurrence and metastases (93).

Many studies have assessed ctDNA prior to and after CRC resection and determined its clinical utility in detecting recurrence. Ryan et al (*n* = 78) contradicted some of the studies above with their finding that pre-operative KRAS2 mutations in ctDNA was not an independent prognostic factor for disease recurrence. However, they did find that KRAS2 ctDNA was positive in patients after surgery and preceding recurrence, which occurred a median of 4 months before CEA elevations (94). Reinert et al had a similar finding with detection of somatic structure variants in post-operative ctDNA an average of 10 months before recurrence in 6 out 9 CRC patients of all stages (95). Several different studies quantified the level of ctDNA from a panel of commonly mutated genes to assess prognosis. In a small study of 18 patients Diehl et al found that detection of high levels of ctDNA post-operatively was associated with recurrence, and ctDNA was a better biomarker than CEA (*p* = 0.03) (47). Schøler et al compared post-operative ctDNA with radiological evidence of recurrence in 14/45 patients in the study who relapsed and found that ctDNA was detected an average of 9.4 months before CT scans (85). Kidess et al assessed 38 patients undergoing liver metastectomy along with CRC resection and found that post-operative ctDNA levels anticipated recurrence earlier than conventional tools—CEA and radiological imaging (96). Pre- and post-operative ctDNA levels have also been evaluated for clinical utility in determining survival. Shin et al assessed *KRAS* mutations in 62 stage III/IV CRC patients undergoing surgery and found a higher rate of ctDNA mutation detection in patients with metastases, and that detectable ctDNA *KRAS* mutations correlated with a shorter overall survival (*p* = 0.03) (97).

Several studies have assessed recurrence in CRC patients based on selection of ctDNA mutations following primary tumor tissue analysis, including a study by Ng et al (*n* = 44) who found certain patients were positive pre-operatively, negative post-operatively and then positive again prior to recurrence before any clinical or radiological evidence (85). Tie et al found that post-operative ctDNA was predictive of recurrence in both locally advanced rectal cancer patients (*n* = 159) (98) and in stage II CRC (*p* = 0.001) (*n* = 178) (99). These findings were irrespective of adjuvant therapy.

Research has also been conducted on patients undergoing chemotherapy. Studies evaluated pre-therapy ctDNA and longitudinal ctDNA collection during treatment and its prognostic role in predicating survival. In 97 metastatic CRC patients, it was shown that high level of cfDNA and high mutation loads of *KRAS* exon2, *BRAF* V600E in pre-therapy ctDNA was associated with shorter overall survival (100). Similar results were also obtained by Spindler et al, detection of *KRAS* mutation in ctDNA correlated with shorter overall survival and progression free survival (*p* = 0.001; *p* = 0.002) in a sample of 140 patients with chemotherapy resistant metastatic CRC (101). Janku and colleagues longitudinally assessed advanced CRC patients receiving chemotherapy in four different studies. In 62 patients receiving BRAF/MEK inhibitors, detection of a high percentage of *BRAF* V600 ctDNA was associated with shorter overall survival and time to failure (*p* = 0.005; *p* = 0.045) (102). In another study of 71 patients, detection of >6.2% *KRAS* G12/13 ctDNA was correlated with shorter survival (*p* = 0.001) (103). Additionally, in a similar study with a cohort of advanced cancer patients (68 colorectal and 3 gastroesophageal), detection of >1% *KRAS, EGFR, BRAF*, or *PIK3CA* mutant ctDNA was associated with a shorter median survival (104). They also tested detection of 61 cancer related genes in 14 CRC patients and found that patients with low variant allele frequency survived longer and the time to treatment failure was also longer (*p* = 0.018; *p* = 0.03). Another important finding in this study was that the allele frequency in patients receiving systemic therapy changed in synchronization with radiological response (*p* = 0.02) (105).

A few studies also looked specifically at using ctDNA as a tool for treatment monitoring and assessing prognosis of metastatic CRC. In a study of 211 patients, Spindler et al found that patients with *KRAS* mutations in pre-therapy ctDNA, did not respond to second-line irinotecan treatment and had shorter overall survival and progression free survival (*p* = 0.04; *p* < 0.0001; *p* = 0.01) (106). In another study of 140 patients, Spindler et al found that pre-therapy DNA levels strongly correlated with *KRAS* ctDNA levels and this was associated with poor disease control using third-line treatment with cetuximab and irinotecan (*p* = 0.009) (107). Tie et al assessed mutations in primary tumor present in ctDNA in 53 patients and found that the changes in level of mutant DNA correlated with radiological response to first-line chemotherapy treatment and major reductions in ctDNA seemed to be associated with a trend for increased progression free and overall survival (108).

### DNA methylation

Methylation changes of certain genes has been investigated by many studies to determine prognosis in different patient groups undergoing surgical resection and/or chemotherapy. There is interest in whether methylated ctDNA markers parallel those using mutations, and one study showed a significant correlation between the two, with both being detectable prior to clinical signs of recurrence (109). Liu et al (*n* = 165) found a significant association between pre-operative ctDNA methylation of *SST* and *MAL* and cancer specific deaths. Methylation of *SST* also correlated with tumor recurrence (31).

Several studies have shown prognostic value of methylated DNA markers. Matthaios et al (*n* = 155) found an association between methylation of *APC* and *RASSF1A* in pre-operative ctDNA and poor survival in early and advanced CRC patients (110). A study of 397 CRC patients under surveillance, assessed accuracy of a panel of methylated ctDNA biomarkers (*BCAT1* and *IKZF1*) and found that sensitivity and specificity for recurrence was 68 and 87% respectively, significantly higher than sensitivity of CEA (32%) with no significant difference in specificity (94%) (37). While most studies have assessed hypermethylation, one study (*n* = 95) found that hypomethylation of *CBS* promoter induced by folate deficiency was also linked to recurrence and cancer-related death (111).

Several studies have investigated the prognostic value of DNA methylation for metastatic CRC patients and/or following adjuvant chemotherapy. Prior to therapy, two studies (*n* = 467 and *n* = 82) showed that detection of methylated *HPP1, WIF1*, and *NPY* in blood have been shown to be associated with poor overall survival (112, 113). The second study showed that a decrease in ctDNA during chemotherapy was associated with longer median progression-free survival and overall survival (*p* < 0.001; *p* < 0.001) (113). Methylation of 30 gene promoter regions was assessed by Rasmussen et al in 193 patients prior to receiving chemotherapy, and a higher number of methylated regions was correlated with an increased risk of metastases. *RARB* and *RASSF1A* methylation was associated with more aggressive disease indicating poor survival (114). In two separate studies Philipp et al (*n* = 311 and *n* = 259) showed that methylation of *HLTF* or *HPP1* was associated with larger and more advanced CRC stage, shorter overall survival and metastases (115, 116).

### Summary

There have been a larger number of studies performed in CRC patients with survival as the key outcome compared to recurrence (Table 5). Recurrence in cases with early stage CRC is a particular challenge for finding prognostic markers that justify individualized therapy aimed at reducing the chance of recurrence. The majority of studies searching for prognostic ctDNA biomarkers for CRC focussed on DNA mutations, with the use of blood biomarkers that have been personalized from primary tumor tissue analysis, showing promising sensitivity. Such biomarkers are most effective though when based on known mutations in surgically resected cancer. Methylated DNA biomarkers are better suited for pre-operative prognostication and hence have been the subject of more studies of this type, with pre-operative detection of methylated *SST* showing promise for independent prediction of recurrence, and methylated *SST, RASSF1A*, and *RARB* being independent predictors of overall survival. More studies are warranted in this field.

**Table 5:** Accuracy of circulating tumor biomarkers for colorectal cancer recurrence and survival (excluding duplicate studies and those with unclear number or fewer than 20 recurrences or deaths).

**RECURRENCE**											
**Author, year**	**Biomarkers**	**Stage**	**Pre-op, post-op or time of recurrence**	**No. recurrence/ total**	**Sensitivity for recurrence**	**Specificity for recurrence**	**NPV for recurrence**	**PPV for recurrence**	**HR for recurrence (95% CI)**	**Adjusted HR (95% CI)**	**Mean/ Median recurrence-free survival**
Ng; 2017 (117)	Mutations: based on tumor tissue findings	All stages (with mutations identified in patient tumor tissue prior to plasma testing)	Post-op; at recurrence	26/44	73%; 96%	83%	N/A	N/A	N/A	N/A	N/A
Ryan; 2003 (94)	Mutation: *KRAS2*	All stages	Pre-op; Post-op	Pre: 20/123 Post: 20/94	52.6%	92%	N/A	62.5%	Pre: 2.07 (0.3-14.8) Post (in stage I-III): 6.37 (2.30-18.0)	N/A	N/A
Tie; 2016 (99)	Mutations: based on tumor tissue findings	Stage II, no chemo (with mutations identified in patient tumor tissue prior to plasma testing)	Post-op	27/178	48%	100%	90.2%	78.6%	18 (7.9-40)	28 (11-68)	N/A
Wang; 2004 (93)	Mutations: *APC, p53, KRAS*	All stages	Pre-op	31/104	87%	81%	91%	75%	N/A	N/A	N/A
Liu; 2016 (31)	Methylation: *SST, MAL, TAC1, SEPT9, EYA4, CRABp1, NELL1*	Stage I-III	Pre-op	43/150	N/A	N/A	N/A	N/A	*SST*: 2.40 (1.27-4.55) *MAL*: 1.15 (0.63-2.09) *TAC1*: 0.96 (0.53-1.74) *SEPT9*: 0.77 (0.42-1.40) *EYA4*:1.18 (0.65-2.15) *CRABp1*: 1.15 (0.63-2.10) *NELL1*:0.73 (0.37-1.44)	*SST*: 2.60 (1.37–4.94) All others: not significant	*SST:* 18.7% (vs. 38.7%), *p* = 0.005
Young; 2016 (37)	Methylation: *BCAT1, IKZF1*	All stages	At recurrence	28/122	68%	87%	N/A	N/A	N/A	N/A	N/A
Xue; 2017 (111)	Hypomethylation: *CBS*	All stages	Pre-op	43/95	74.4%	59.6%	74%	62%	1.62 (1.29-3.68)	1.54 (1.18-3.02)	N/A
**SURVIVAL**											
**Author, year**	**Biomarkers**	**Stage**	**Pre-op or post-op**	**No. deaths/total**	**Sensitivity for death**	**Specificity for death**	**NPV for death**	**PPV for death**	**HR for poor survival (95% CI)**	**Adjusted HR (95% CI)**	**Mean/ Median survival**
Schwarzenbach; 2008 (83)	DNA level (spectrophotometric quantification at 260 and 280nm)	Stage IV	Pre-op	33/55	N/A	N/A	N/A	N/A	N/A	N/A	Association with shorter survival (*p* = 0.02)
Li; 2017 (118)	cfDNA copy number variation	Stage III-IV	Post-op	23/35	N/A	N/A	N/A	N/A	5.33 (6.76-94.44)	N/A	15.87 months (vs. 68.53 months)
Bedin; 2017 (84)	DNA fragments (Alu83 and Alu244); Methylation: *OSMR, SFRP1*	All stages	Pre-op	28/114	N/A	N/A	N/A	N/A	Alu83: 3.49 (1.58-7.71) Alu244: 2.70 (1.25-5.84)	Alu83: 2.71 (1.22-6.02) Alu244: 2.40 (1.11-5.19)	Methylation: No association with survival
Lin; 2014 (90)	DNA copy number (cyclophilin); Mutations: 74 genes (including *KRAS, APC, TP53, PIK3CA, BRAF*)	All stages	Pre-op	62/191	N/A	N/A	N/A	N/A	DNA copy number: 3.25 (1.66-6.45) Mutations: p>0.05	DNA copy number: 2.61 (1.31-5.19)	DNA copy number: 43% (vs. 78%), *p* = 0.001 Mutations: 48.8% (vs. 77%), *p* = 0.008
Tie; 2015 (108)	Mutations: based on tumor tissue findings (including *KRAS*)	Stage IV (with mutations identified in patient tumor tissue prior to plasma testing)	Post-op	20/53	N/A	N/A	N/A	N/A	N/A	N/A	No association between change in ctDNA and survival
Herbst; 2017 112)	Methylation: *HPP1*	Stage IV (treated with a combination therapy containing a fluoropyrimidine, oxaliplatin, and bevacizumab)	Pre-op	246/467	N/A	N/A	N/A	N/A	1.86 (1.37-2.35)	N/A	21.9 months (vs. 35.2 months)
Liu; 2016 (31)	Methylation: *SST, MAL, TAC1, SEPT9, EYA4, CRABp1, NELL1*	All stages	Pre-op	58/165	N/A	N/A	N/A	N/A	*SST*: 2.40 (1.35-4.28) *MAL*: 2.26 (1.29-3.96) *TAC1*: 1.15 (0.67-1.97) *SEPT9*: 1.02 (0.59-1.74) *EYA4*:1.24 (0.73-2.12) *CRABp1*: 1.10 (0.64-1.87) *NELL1*:1.19 (0.68-2.07)	*SST*: 1.96 (1.06-3.62) All others: not significant	*SST* in stage II and III 16.1% (vs. 41.9%), *p* = 0.003
Philipp; 2012 116)	Methylation: *HLTF, HPP1*	All stages	Pre-op	190/311	N/A	N/A	N/A	N/A	N/A	N/A	*HLTF:* 36.3 months (vs. 80.2 months), *p* = 0.0001 *HPP1:* 12.6 months (104.7 months), *p* < 0.0001
Rasmussen; 2018 (114)	Methylation: *ALX4, BNC1, HIC1, RARB, RASSF1A, SDC2, SEPT9, SRFP1, SRFP2, SPG20, TFP12, THBD, WIF1, APC, BMP3, BRCA1, CDKN2A, HLTF, MGMT, MLH1, NDRG4, NPTX2, NEUROG1, OSMR, PHACTR3, PPENK, SST, TAC1, VIM, WNT5A*	All stages	Pre-op	74/193	N/A	N/A	N/A	N/A	*ALX4:* 2.18 (1.41-3.38) *BNC1:* (2.93 (1.69-5.07) *HIC1:* 3.04 (1.46-6.32) *RARB:* 2.06 (1.31-3.23) *RASSF1A:* 2.75 (1.57-4.81) *SDC2:* 1.94 (1.23-3.07) *SEPT9:* 1.91 (1.21-3.02) *SRFP1:* 1.91 (1.21-3.02) *SRFP2:* 2.42 (1.53-3.84) *SPG20:* 2.66 (1.67-4.24) *TFP12:* 2.67 (1.38-5.19) *THBD:* 2.95 (1.68-5.18) *WIF1:* 3.26 (1.83-5.83) *HLTF:* 1.87 (1.04-3.38) *MHMT:* 2.27 (1.04-4.93) *PPENK:* 1.94 (1.07-3.50) *TAC1:* 1.56 (1.01-2.42) *VIM:* 1.82 (0.84-3.96) All others: not significant	*RARB:* 1.99 (1.07-3.72) *RASSF1A:* 3.35 (1.76-6.38) All others: not significant	N/A
Xue; 2017 (111)	Hypomethylation: *CBS*	All stages	Pre-op	37/95	75.7%	56.9%	80%	53%	1.49 (1.15-2.64)	1.35 (1.09-2.41)	N/A

*CI, Confidence interval; HR, hazard ratio; N/A, not applicable; NPV, negative predictive value; PPV, positive predictive value; post-op, post-operative; pre-op, pre-operative*.

## Discussion

Following cancer diagnosis, clinical decisions regarding treatment and surveillance frequency are largely driven by pathological stage. Despite this there are a considerable proportion of patients who still have cancer recurrence and poor survival. Non-invasive biomarkers that can provide an accurate prognosis assessment independent of stage are therefore warranted. While there have been a large number of studies conducted in gastrointestinal cancers, the majority have assessed prognosis for CRC. Very few studies report diagnostic accuracy for either recurrence or death (sensitivity and specificity), and many are limited by small numbers of patients with endpoints of recurrence or mortality. In addition, out of all of the studies reviewed (when limiting analysis to those studies with at least 20 events of interest), very few ctDNA biomarkers are independent predictors of recurrence or survival. For oesophageal cancer and GIST there were no independent biomarkers for prognosis. For gastric cancer methylated *SOX17* and *APC* were independent predictors of survival, with an adjusted HR of 3.0 (95% CI 1.2–7.8) (72) and 4.6 (95% CI 1.1–20.3) (71) respectively. For CRC there were a number of ctDNA biomarkers that were independent predictors of prognosis including DNA levels and fragments, tumor-specific DNA mutations and DNA methylation. A personalized ctDNA panel based on tumor tissue analysis gave the greatest independent prediction of recurrence with a HR of 28 (95% CI 11–68) (99). Other independent predictors for recurrence included methylated *SST* (HR 2.60, 95% CI 1.37–4.94) (31) and hypomethylated *CBS* (HR 1.54, 95% CI 1.18–3.02) (111). For independent prediction of survival, seven potential biomarkers (all analyzed in pre-operative blood samples) were found: Alu83 (HR 2.71, 95% CI 1.22–6.02) (84), Alu244 (HR 2.70, 95% CI 1.25–5.84) (84), DNA copy number (HR 2.61, 95% CI 1.31–5.19) (90), methylated *SST* (HR 1.96, 95% CI 1.06–3.62) (31), methylated *RARB* (HR 1.99, 95% CI 1.07–3.72) (114), methylated *RASSF1A* (HR 3.35, 95% CI 1.76–6.38) (114), and hypomethylated *CBS* (HR 1.35, 95% CI 1.09–2.41) (111). As can be seen, in most cases the reported hazard ratios for prognosis were not stronger than those found with the clinicopathological variables reported in Table 1.

### Limitations in studies of ctDNA

In this review we have not taken into consideration the methodological differences between studies which can affect results, leading to false positives or negatives. Variations in blood collection tubes, storage times, and temperatures, DNA isolation methods, and nature of analysis (automated or manual) are all relevant to assessing benefit. One study that compared different blood collection tubes for analysis of epigenetic alterations in ctDNA found that some could only be stored cold for 24 h, while others could be stored at room temperature for 48 h (119). In addition, the use of plasma or serum can introduce differences in results. Serum typically has higher yields of DNA (85, 120), but this may be from contamination of the sample with DNA from white blood cells, which lyse during serum processing (120). A study showed that DNA levels from serum and plasma did not correlate. Serum DNA was associated with the presence of liver metastases, while only DNA from plasma was predictive for recurrences (121). Another study showed that serum samples compared to plasma samples had a decreased *KRAS* allele frequency (122). This suggests that plasma is the optimal specimen type for analysis of ctDNA (123), but despite this, approximately one-third of the studies that we reviewed had used serum (20% of CRC studies, 29% of oesophageal cancer studies, 62% of gastric cancer studies, and 100% of GIST studies).

Other features that need to be considered for ctDNA studies are amplicon lengths and time of collection. As circulating DNA is highly fragmented, targeted regions of the DNA need to account for this. By using a short amplicon assay, KRAS mutated DNA was detected in significantly more blood samples compared to using a long amplicon assays (124). Time of blood collection may also influence levels of ctDNA, as it has been shown that total DNA and levels of methylated Septin 9 (*SEPT9*) have diurnal variations (125). In patients with CRC, highest concentrations were measured at midnight (125).

All studies, whether of mutations or methylation markers, are subject to the chance that detection of biomarkers might not be associated with the tumor of interest. This was supported by a study of *TAC1* hypermethylation in oesophageal cancer which found that ~13% of their cohort had the biomarker present in plasma but not in the matched tumor tissue (61). They proposed that this could indicate a risk for developing malignant disease in the future; that it could be derived from a pre-cancerous lesion; or it could be derived from a cancer elsewhere in the body. It is possible that ctDNA biomarkers may not be specific to just one cancer. While hypermethylation of the promoter region of *SEPT9* shows promise for screening and monitoring of CRC, methylated *Sept9* was also detected in 44.3% of lung cancer patients (126). In the current review, the lack of specificity for one cancer was seen for methylated *APC* and *RASSF1A* that have prognostic potential in both gastric (70, 71) and colorectal cancers (110, 114). These studies highlight the importance of optimization of ctDNA assays.

### Choice of biomarker

Many of the studies of prognosis have used DNA mutations as ctDNA biomarkers. Due to tumor heterogeneity, assessment of mutations is not easily implemented in practice, with the common genes (*KRAS, BRAF, APC, TP53*) mutated in only 15–40% of CRC (127). This is why several of the studies that we reviewed applied tumor tissue analysis to personalize ctDNA biomarkers. Extensive analysis of tumor prior to blood may reduce cost effectiveness of the liquid biopsy, and in addition, this limits the ctDNA biomarkers to assessment of certain tumor subtypes rather than being a universal marker of prognosis. Measurement of DNA methylation may be an easier test to apply. Methylated DNA is present in a higher proportion of tumors than mutations, for example 82% of primary tumor tissue displays *SEPT9* promoter methylation (128). There is also evidence that aberrant methylation is more common and frequently precedes the mutational changes (53). The consequences of promoter methylation can include transcriptional silencing which might facilitate tumor progression by allowing the accumulation of additional genetic and/or epigenetic changes (129). As the metastatic capacity of a cell is determined at an early stage of tumor progression (130) it seems possible to identify epigenetic biomarkers that point to tumor aggressiveness.

### Other clinical management strategies for ctDNA

Besides its use for prognosis, there is a lot of interest in the use of ctDNA in relation to treatment strategies. As ctDNA provides real-time results that reflect the current molecular profile of the tumor tissue which are likely to be more representative of the entire tumor rather than a single biopsy (131), ctDNA results could be used to plan appropriate therapy. Analysis of ctDNA from patients with gastrointestinal malignancies showed that most of the patients tested had one or more alterations potentially actionable by experimental or approved drugs (132). ctDNA can also be used in monitoring treatment efficacy with one such example shown with changes of ctDNA *HER2* copy number with trastuzumab treatment in gastric cancer (133). In relation to monitoring efficacy, ctDNA also shows value in detecting the development of secondary resistance to therapy. Examples have been shown in both CRC and gastric cancer with ctDNA detecting growth of mutated clones. For example, in patients with metastatic CRC, *RAS* mutations emerged during therapy with anti-EGFR mAB which indicated resistance (134). Similarly use of serial ctDNA measurements have shown emerging resistance to crizotinib use in gastric cancer (135). It is also possible that the detection of new mutations with ctDNA, or detection of a number of biomarkers identifies tumor heterogeneity, and indicates prognosis as well as guiding therapy. Clinical use of ctDNA for monitoring of therapies will allow the use of ineffective therapies to be ceased earlier. The use of ctDNA for metastatic CRC is supported by physicians, with 69% of physicians reporting that it was more convenient than tissue testing, 59% believing ctDNA to be the superior method to guide experimental therapy choice, and reporting that 89% of their patients were satisfied with the ability of this method to improve quality of care (136).

## Conclusion

Application of new strategies for prognostication and personalized management are needed to improve survival from gastrointestinal cancers. This can be achieved with ctDNA. Due to heterogeneity of disease, single biomarkers are less likely to have sufficient sensitivity and specificity and therefore a combination of biomarkers and techniques could maximize diagnostic accuracy. Our review shows that the use of ctDNA shows great promise as prognostic biomarkers for recurrence and survival, however caution should be taken with interpreting results from studies with limited sample sizes. As well as prognostication, markers might allow early detection of recurrence. This will result in survival benefits from resection when lesions are treatable, as well as permitting earlier commencement of therapy.

## Author contributions

ES and GY came up with the manuscript concept. HS and ES reviewed all of the articles and drafted the manuscript. GY and CK contributed clinical advice. SP contributed molecular advice. GY, CK, and SP thoroughly revised and amended the manuscript.

### Conflict of interest statement

SP is employed by Clinical Genomics Pty Ltd and GY is a paid consultant for Clinical Genomics Pty Ltd. No funding was received in relation to this manuscript. The remaining authors declare that the research was conducted in the absence of any commercial or financial relationships that could be construed as a potential conflict of interest.
