# Supplementary material for: The Use of Circulating Tumor DNA for Prognosis of Gastrointestinal Cancers
Source: Front Oncol. 2018 Jul 24;8:275. doi: 10.3389/fonc.2018.00275 (PMC6066577; doi:10.3389/fonc.2018.00275)
Supplement: Supplementary file 1 [file Table_1.docx]

**Supplementary Table 1. Studies that have assessed circulating tumour DNA (ctDNA) for recurrence and death for oesophageal tumours.**

| ***Recurrence*** | | | | | | | | | | |
| --- | --- | --- | --- | --- | --- | --- | --- | --- | --- | --- |
| **Author, year** | **Sample size** | **Cancer stage** | **Age (y, median or mean, range)** | **% Male** | **Biomarker** | **Method** | **Time of sample collection** | **Follow-up time** | **Comparison to clinically used methods** | **Finding** |
|  | **No. with recurrence** |  |  |  |  |  |  |  |  |  |
| Eisenberger, CF; 2003 (1) | 28 | Squamous cell carcinoma; all stages | 62y (37-74y) | NA | 12 microsatellite markers that indicate LOH (D17S520, D17S796, D17S 804, D9S162,  D9S171, D9S1748, D9S126, D18S51,  D18S70, ACTBP2, CSF1R, D18S479; related to *p16, p53, DPC4* and *APC,*) | Serum, PCR | Pre-surgery | Median 13 months | No | LOH in serum was not associated with recurrence. |
|  | 11? |  |  |  |  |  |  |  |  |  |
| Eisenberger, CF; 2006 (2) | 32 | Adenocarcinoma; all stages | 61y (34-78y) | NSNA | 12 microsatellite markers that indicate LOH (D17S520, D17S796),  D17S 804, D9S162, D9S171, D9S1748, D9S126, D18S51, D18S70, ACTBP2, CSF1R, D18S479; related to *p16, p53, DPC4* and *APC,*) | Serum, PCR | Pre-surgery | 2-60 months (median 18 months) | No | LOH in serum was not associated with recurrence. |
|  | 22 |  |  |  |  |  |  |  |  |  |
| Hseih, CC; 2016 (3) | 81 | Squamous cell carcinoma; stage I-III | 60y (38-84y) | 86% | DNA copy numbers (cyclophilin) | Plasma, PCR | Post-surgery | ≤120 months | No | Higher DNA level was associated with higher incidence for recurrence (p=0.018) |
|  | 49 |  |  |  |  |  |  |  |  |  |
| Ueda, M; 2016 (4) | 13  (follow-up n=4) | Squamous cell carcinoma; all stages | 67y (53-79y) | 92% | Mutations in 53 cancer-related genes (including *TP53, FAT3, MLL3, AJUBA*) | Plasma; NGS | Pre-surgery and post-surgery, follow-up for n=4 | Up to 15 months | *p53* antibody and squamous cell carcinoma (SCC)-related antigen | In 4 with follow-up, 2 had recurrence and allele frequency of mutations increased 6-9 months before recurrence. *p53* and SCC-related antigen remained within the normal range. |
|  | 3 |  |  |  |  |  |  |  |  |  |
| ***Survival*** | | | | | | | | | | |
| **Author, year** | **Sample size** | **Cancer stage** | **Age (y)** | **% Male** | **Biomarker** | **Method** | **Sampling time** | **Follow-up time** | **Comparison to clinically used methods** | **Finding** |
|  | **No. deaths** |  |  |  |  |  |  |  |  |  |
| Eisenberger, CF; 2006 (2) | 32 | Adenocarcinoma; all stages | 61y (34-78y) | NA | 12 microsatellite markers that indicate LOH (D17S520, D17S796, D17S 804, D9S162,  D9S171, D9S1748, D9S126, D18S51,  D18S70, ACTBP2, CSF1R, D18S479; related to *p16, p53, DPC4* and *APC,*) | Serum, PCR | Pre-surgery | 2-60 months (median 18 months) | No | LOH in serum was not associated with survival. |
|  | 24 |  |  |  |  |  |  |  |  |  |
| Eisenberger, CF; 2003 (1) | 28 | Squamous cell carcinoma; all stages | 62y (37-74y) | NA | 12 microsatellite markers that indicate LOH (D17S520, D17S796),  D17S 804, D9S162, D9S171, D9S1748, D9S126, D18S51, D18S70, ACTBP2, CSF1R, D18S479; related to *p16, p53, DPC4* and *APC,*) | Serum, PCR | Pre-surgery | Median 13 months | No | LOH in serum was not associated with survival. |
|  | 9? |  |  |  |  |  |  |  |  |  |
| Hseih, CC; 2016 (3) | 81 | Squamous cell carcinoma; stage I-III | 60y (38-84y) | 86% | DNA copy numbers (cyclophilin) | Plasma, PCR | Post-surgery | ≤120 months | No | Higher DNA level was associated with poorer overall survival but this was not significant (p=0.164) |
|  | 43 |  |  |  |  |  |  |  |  |  |
| Hoffmann, AC; 2009 (5) | 59 | Squamous cell carcinoma and adenocarcinoma; all stages | 59y | 85% | Methylation: *DAPK* and *APC* (corrected for β-actin) | Plasma, PCR | pre-surgery and 10 day post-surgery | ≤60 months | No | Pre-surgical plasma positive for methylated *DAPK* predicted those with poorer survival with a difference more than 40 months in survival (vs no methylation). This was independent of TNM stage (p=0.012). Methylated *APC* was not associated with survival. |
|  | NA |  |  |  |  |  |  |  |  |  |
| Jin, Z; 2007 (6) | 61 | Adenocarcinoma; all stages | NA (approx. 65y) | NA | Methylation: *Tac1* (corrected for β-actin) | Plasma, PCR | Pre-surgery | ≤180 months | No | Presence of methylated *Tac1* in plasma was not associated with survival. |
|  | NA |  |  |  |  |  |  |  |  |  |
| ***Disease-free survival (recurrence + death)*** | | | | | | | | | | |
| **Author, year** | **Sample size** | **Cancer stage** | **Age (y)** | **% Male** | **Biomarker** | **Method** | **Time of sample collection** | **Follow-up time** | **Comparison to clinically used methods** | **Finding** |
|  | **No. with recurrence ± death** |  |  |  |  |  |  |  |  |  |
| Ling, ZQ; 2012 (7) | 209  (63 had follow-up) | Squamous cell carcinoma; all stages | 60y (40-85y) | 88% | Methylation: *MSH2* (relative to unmethylated *MSH2*) | Plasma; PCR | Pre-surgery for all, and post-surgery for n=63 | 2-3 months | No | Mortality increased with increase in *MSH2* methylation post-surgery. RR for disease-free survival was 21.4 after correction for sex, age and stage. |
|  | 20 |  |  |  |  |  |  |  |  |  |

LOH: loss of heterozygosity; NGS: next-generation sequencing; NA: not available; RR: risk ratio;

**Table 2. Studies that have assessed ctDNA for recurrence and survival for gastric cancers.**

| ***Recurrence*** | | | | | | | | | | |
| --- | --- | --- | --- | --- | --- | --- | --- | --- | --- | --- |
| **Author, year** | **Sample size** | **Cancer stage** | **Age (y, median or mean, range)** | **% Male** | **Biomarker** | **Method** | **Time of sample collection** | **Follow-up time** | **Comparison to clinically used methods** | **Finding** |
|  | **No. with recurrence** |  |  |  |  |  |  |  |  |  |
| Fang WL; 2016 (8) | 277 | All stages | 69y | 77% | DNA copy numbers (cyclophilin), mutations: *ARID1A, TP53, PIK3CA, PTEN, AKT3, BRAF, AKT1, AKT2* | Plasma, PCR | Pre-surgery | 61 months (2-232 months) | No | Disease free survival did not differ between cases with low or high DNA levels (p=0.175). |
|  | 129 |  |  |  |  |  |  |  |  |  |
| Hamakawa T; 2015 (9) | 42 (follow-up n=3) | Stage III and IV with *TP53* mutation in tumour | 80y | 100% | DNA copy numbers (LINE-1), mutations: *TP53, PIK3CA, FBXw7* | Plasma, deep sequencing PCR | Pre-surgery and follow-up | NA | CEA, CA 19-9 | Levels of mutation ctDNA rose with recurrence, matching with CEA and CA 19-9, but DNA levels did increase. |
|  | 3 |  |  |  |  |  |  |  |  |  |
| Lan, YT; 2017 (10) | 428  (follow-up n=18) | All stages | 66y | 72% | DNA copy numbers (cyclophilin) | Serum, PCR | Pre-surgery (all), post-surgery and time of recurrence (n=18) | Approx. 18 months | No | Cases with recurrence had high DNA copy number post-surgery (but not clear what levels are in non-recurrence cases) |
|  | 18 |  |  |  |  |  |  |  |  |  |
| Pu, WY; 2016 (11) | 73 | All stages | 62y (18-78y) | 63% | DNA fragment lengths (*Alu219*, *Alu115*) | Plasma, PCR | Pre-therapy (all), 1-3wk post-surgery, and during follow-up (n=11) | 24 months | No | There was no correlation between disease-free survival and pre-surgery *Alu115* (p=0.95) |
|  | 11 |  |  |  |  |  |  |  |  |  |
| Shoda K; 2017 (12) | 60 | Stage I-III (with tissue *HER2* positive and negative) | NA | 73% | *HER2* copy number (relative to *RPPH1*) | Plasma, digital droplet PCR | pre-surgery; 1 month post-surgery (n=21); at recurrence (n=17) | ≤ 23 months | CEA, CA 19-9 | There were high post-operative plasma *HER2* at the time of recurrence in 10 of 17 cases. *limited data provided for CEA and CA 19-9. |
|  | 17 |  |  |  |  |  |  |  |  |  |
| Shoda K; 2017 (13) | 153 | Stage I-III (21 with EBV-associated tumours) | NA | 62% | EBV DNA (relative to *RPPH1*) | Plasma, PCR | pre-surgery; 1 month post-surgery (n=9); follow-up (n=1) | 31 months (5-85 months) | CEA, CA 19-9 | There was no significant difference in the recurrence-free survival between the pre-surgery plasma EBV-positive and negative cases. In one case with recurrence, post-surgery samples showed an increase in EBV DNA 1 month before recurrence, but no elevation in CEA or CA 19-9. |
|  | NA |  |  |  |  |  |  |  |  |  |
| ***Survival*** | | | | | | | | | | |
| **Author, year** | **Sample size** | **Cancer stage** | **Age (y, median or mean, range)** | **% Male** | **Biomarker** | **Method** | **Sampling time** | **Follow-up time** | **Comparison to clinically used methods** | **Finding** |
|  | **No. deaths** |  |  |  |  |  |  |  |  |  |
| Balgkouranidou I; 2015 (14) | 73 | Operable cancer- stages I-III | 67y | 66% | Methylation: *APC* and *RASSF1A* (relative to unmethylated levels) | Serum, PCR | Pre-surgery | 56 months (12-111 months) | CEA, CA 19-9 | Incidence of death was higher in cases with methylated *APC* compared to no methylation (59.0% vs.  16.7%, p = 0.007), survival time was shorter (46 months vs 85 months) and it was an independent predictor of poor survival (HR 4.6). Levels of *APC* methylation correlated with CEA and CA 19-9. Incidence of death was not related to *RASSF1A* methylation (50.0% vs 56.5% with no methylation  p = 0.604). |
|  | 38 |  |  |  |  |  |  |  |  |  |
| Balgkouranidou I; 2013 (15) | 73 | Operable cancers | 71y (28-82y) | 66% | Methylation: *SOX17* (relative to unmethylated levels) | Serum, PCR | Pre-surgery | Median 56 months (20-111 months) | No | *SOX17* methylation was associated with a reduction in survival compared to no methylation (37.7 vs 66.9 months). Methylation was an independent predictors of poor survival (HR=3.0). Sensitivity for poor survival was 68%, specificity was 51%. |
|  | 38 |  |  |  |  |  |  |  |  |  |
| Fang WL; 2016 (8) | 277 | All stages | 69y | 77% | DNA copy numbers (cyclophilin), mutations: *ARID1A, TP53, PIK3CA, PTEN, AKT3, BRAF, AKT1, AKT2* | Plasma, PCR | Pre-surgery | 61 months (2-232 months) | No | Overall survival did not differ between cases with low or high DNA levels (p=0.291). For stage III and IV cancers with DNA mutations, there was a lower 5y survival rate compared to no mutations (5.6% vs 31.5%). No differences were found for stage I and II. |
|  | 179 |  |  |  |  |  |  |  |  |  |
| Kinugasa H; 2015 (16) | 25 | Stage III and IV (with tissue *HER2* positive and negative, and directed therapy to *HER2* positive cases) | 66y (29-81y) | 80% | *HER2* copy numbers (relative to *EFTUD2*) | Serum, digital droplet PCR | Pre-surgery/ treatment | 20 months | No | *HER2* ctDNA positive cases (3/7 treated with trastuzumab) had a shorter survival compared to *HER2* negative (124 days vs 321 days, p=0.01). |
|  | NA |  |  |  |  |  |  |  |  |  |
| Pimson C; 2016 (17) | 101 | All stages | NA | 56% | Methylation: *PCDH10* and *RASSF1A* (relative to unmethylated levels) | Plasma, PCR | Pre-surgery | 72-120 months | No | *RASSF1A* methylation was associated with a reduction in survival compared to no methylation (7.8 vs 20.2 months). *PCDH10* methylation had a survival of 8.4 months compared to no mortality. *RASSF1A* methylation and stage were independent predictors of survival. |
|  | NA |  |  |  |  |  |  |  |  |  |
| Shoda K; 2017 (13) | 153 | Stage I-III (21 with EBV-associated tumours) | NA) | 62% | EBV DNA (relative to *RPPH1*) | Plasma, PCR | pre-surgery; 1 month post-surgery (n=9); follow-up (n=1) | 31 months (5-85 months) | CEA, CA 19-9 | There was no significant difference in the overall-free survival between the pre-surgery plasma EBV-positive and negative cases. |
|  | NA |  |  |  |  |  |  |  |  |  |
| ***Disease-free survival (recurrence + death)*** | | | | | | | | | | |
| **Author, year** | **Sample size** | **Cancer stage** | **Age (y, median or mean, range)** | **% Male** | **Biomarker** | **Method** | **Time of sample collection** | **Follow-up time** | **Comparison to clinically used methods** | **Finding** |
|  | **No. with recurrence ± death** |  |  |  |  |  |  |  |  |  |
| Han J; 2014 (18) | 92 | All stages | NA | 58% | Methylation: *MINT2* (relative to unmethylated levels) | Serum, PCR | Pre-surgery | ≤ 40 months | No | Cases with methylated *MINT2* developed recurrence more than 32 months earlier than those without methylation. Methylation was an independent predictor disease-free survival (RR 4.1) |
|  | NA |  |  |  |  |  |  |  |  |  |
| Ling ZQ; 2013 (19) | 202 | All stages | NA | 59% | Methylation: *XAF1* (relative to unmethylated levels) | Serum, PCR | Pre-surgery (all), 1-6 months post-surgery (n=72) | ≤ 40 months | No | Cases with *XAF1* methylation in serum had a lower disease free survival compared to those without methylation. It was an independent predictor (HR 5.71). Post-surgical increases in methylation were associated with recurrence. |
|  | 118 |  |  |  |  |  |  |  |  |  |
| Yu JL; 2014 (20) | 92 | All stages | NA | 59% | Methylation: *TIMP-3* (relative to unmethylated levels) | Serum, PCR | Pre-surgery | ≤ 40 months | No | Survival was more than 26 months less in those with methylated *TIMP3*. Methylation was an independent predictor (HR 1.55). |
|  | NA |  |  |  |  |  |  |  |  |  |

HR: hazard ratio; NA: not available; RR: relative risk

**Table 3. Studies that have assessed ctDNA for recurrence and survival for gastrointestinal stromal tumours.**

| ***Recurrence*** | | | | | | | | | | | |
| --- | --- | --- | --- | --- | --- | --- | --- | --- | --- | --- | --- |
| **Author, year** | **Sample size** | **Cancer stage** | **Age (y, median or mean, range)** | **% Male** | **Biomarker** | **Method** | **Time of sample collection** | | **Follow-up time** | **Comparison to clinically used methods** | **Finding** |
|  | **No. with recurrence** |  |  |  |  |  |  |  |  |  |  |
| Rawnaq T; 2011 (21) | 101 | Advanced or recurrent GIST | 59% were >55y | 52% | 12 microsatellite markers for LOH (D22S683, D22S689, D22S685, D22S446, D22S445,  D22S425, D17S804, D17S796, D17S520,  D13S262, D9S162, D3S1286; related to *NF2, VHL, p16, Rb, p53*) | Serum; PCR | Follow-up | | Median 49 months | No | ≥2 LOH was detected in 58% of recurrent cases |
|  | 29 |  |  |  |  |  |  |  |  |  |  |
| ***Survival*** | | | | | | | | | | | |
| **Author, year** | **Sample size** | **Cancer stage** | **Age (y, median or mean, range)** | **% Male** | **Biomarker** | **Method** | | **Sampling time** | **Follow-up time** | **Comparison to clinically used methods** | **Finding** |
|  | **No. deaths** |  |  |  |  |  |  |  |  |  |  |
| Rawnaq T; 2011 (21) | 101 | Advanced or recurrent GIST | 59% were >55y | 52% | 12 microsatellite markers for LOH (D22S683, D22S689, D22S685, D22S446, D22S445,  D22S425, D17S804, D17S796, D17S520,  D13S262, D9S162, D3S1286; related to *NF2, VHL, p16, Rb, p53*) | Serum; PCR | | Follow-up | Median 49 months | No | ≥2 LOH had poorer overall survival, but not significantly different |
|  | NA |  |  |  |  |  |  |  |  |  |  |
| Yoo C; 2014 (22) | 27 | Tyrosine kinase  inhibitor-refractory | 35-76y | 67% | Mutations: 29 in *KIT*,  5 in *PDGFRα*,  1 in *BRAF* | Serum; BEAMing | | Day 1 of cycle 1 and 2 | Approx. 12 months | No | Those with a secondary *KIT* mutation had poorer overall survival (5.5 months vs 9.8 months), p=0.047 |
|  | NA |  |  |  |  |  |  |  |  |  |  |

LOH: loss of heterozygosity; NA: not available

**Table 4. Studies that have assessed ctDNA for recurrence and survival for colorectal cancers.**

| ***Recurrence*** | | | | | | | | | | | |
| --- | --- | --- | --- | --- | --- | --- | --- | --- | --- | --- | --- |
| **Author, year** | **Sample size** | **Cancer stage** | **Age (y, median or mean, range)** | **% Male** | **Biomarker** | **Method** | **Time of sample collection** | | **Follow-up time** | **Comparison to clinically used methods** | **Finding** |
|  | **No. with recurrence** |  |  |  |  |  |  |  |  |  |  |
| Cassinotti E; 2013 (23) | 223 | All stages | 66y | 55% | DNA copy number (measured with RNAse P gene) | Plasma, PCR | Pre-surgery, every 3 months post-surgery | | ≤ 60 months | No | In cases with recurrence DNA level increased before clinical evidence. *graphs show 3 examples and only 1 example of non-recurrence. |
|  | NA |  |  |  |  |  |  |  |  |  |  |
| Diehl F; 2008 (24) | 18 | Stages II-IV (with mutations identified in patient tumour tissue prior to plasma testing) | 60y (35-82y) | 44% | Mutations:  *APC, PIK3CA, KRAS, TP53,* (relative to wild-type), and total DNA (*LINE-1*) | Plasma, BEAMing and PCR | Pre-surgery, and 1, 2-10 and 13-56 days post-surgery | | ≤ 24 months | CEA | Risk of recurrence was associated with presence of post-surgery ctDNA mutations (p=0.006), with a greater prediction than CEA. |
|  | 13 |  |  |  |  |  |  |  |  |  |  |
| Frattini M; 2006 (25) | 70 | All stages | NA | NA | DNA level | Plasma, DNA DipStick Kit | Pre-surgery and 4 and 10 months post-surgery | | 10 months | CEA | DNA level increased in cases with recurrence, but decreased in those without recurrence. There was no correlation between DNA levels and CEA concentration. |
|  | NA |  |  |  |  |  |  |  |  |  |  |
| Garrigou S; 2016 (26) | 148 (follow-up in n=9) | All stages | NA | NA | Mutations:  *KRAS, BRAF, TP53, PIK3CA, NRAS* Methylation: *WIF1, NPY, PENK* | Plasma, digital droplet PCR. | Pre-surgery and every 3-5 months post-surgery | | ≤ 36 months | No | ctDNA was detectable prior to clinical detection of recurrence. Methylation and mutation levels correlated. |
|  | 5 |  |  |  |  |  |  |  |  |  |  |
| Guadalajara H; 2008 (27) | 73 | All stages | 68y | 64% | DNA level | Plasma, spectrophotometry (NanoDrop) | Pre-surgery | | 554 days (10-1407 days) | No | There was no association between DNA level and recurrence. |
|  | 8 |  |  |  |  |  |  |  |  |  |  |
| Kidess E; 2014 (28) | 38 (follow-up in n=4) | All stages (follow-up in only stage IV with liver metastasis) | 63y (39-89y) | 61% | Mutations:  *BRAF, EGFR, KRAS, PIK3CA* | Plasma, SCODA | Pre-surgery and multiple times with follow-up | | ≤ 9 months | CEA | Post-surgery ctDNA level predicted recurrence earlier than CEA. *Based on 2 that developed recurrence. |
|  | 4 |  |  |  |  |  |  |  |  |  |  |
| Lan, YT; 2017 (10) | 329 (follow-n=54) | All stages | 69y | 55% | DNA copy number (cyclophilin) | Serum, PCR | Pre-surgery (all), post-surgery (n=54) and at time of recurrence (n=4) | | Approx. 18 months | CEA | Post-surgery DNA level was high in n=4, 50% developed recurrence. CEA was high in n=16, 25% developed recurrence. |
|  | NA (4-6?) |  |  |  |  |  |  |  |  |  |  |
| Lecomte T; 2002 (29) | 37 | All stages (tumours with either *KRAS2* mutation or *p16* gene promoter methylation) | 69y | 50% | Mutation:  *KRAS2*  Methylation: *p16* (relative to unmethylated levels) | Plasma, PCR | Pre-surgery | | Mean 22 months | No | 2y recurrence‐free survival was 66% for cases with ctDNA, compared to 100% without ctDNA (for stages I-III, p=0.044). |
|  | 6 |  |  |  |  |  |  |  |  |  |  |
| Liu Y; 2016 (30) | 165 | All stages | 67y | 55.2% | Methylation:  *SST, MAL, TAC1, SEPT9, EYA4, CRABp1, NELL1* (normalised to β-actin) | Plasma, PCR | Pre-surgery | | Median 56 months | CEA | Cases with methylated *SST* or high CEA were associated with lower disease-free survival. Only methylated *SST* was an independent predictor (HR 2.6). Recurrence was higher in the methylated *SST* group (38.7%) compared to no methylation (18.7%). |
|  | 43 |  |  |  |  |  |  |  |  |  |  |
| Ng SB; 2017 (31) | 44 | All stages (with mutations identified in patient tumour tissue prior to plasma testing) | 61y (45-77y) | 77% | Mutations based on tumour tissue findings | Plasma, PCR | Pre-surgery, post-surgery and follow-up | | ≤ 13 months | CEA | 96% of cases with recurrence had detectable ctDNA. 73% of cases had increased ctDNA prior to recurrence. Not all had increased CEA. |
|  | 26 |  |  |  |  |  |  |  |  |  |  |
| Reinert T; 2016 (32) | 11 | All stages (with mutations identified in patient tumour tissue prior to plasma testing) | 73y | 64% | DNA level and mutations based on tumour tissue findings (including KRAS mutations) | Plasma, NGS, digital droplet PCR | Pre-surgery and multiple times post-surgery | | 36 months | CEA | ctDNA detected recurrence 10 months earlier than CT for all cases. Sensitivity and specificity was 100%. DNA level was increased in 83% of cases at recurrence. CEA had a sensitivity of 67% and specificity of 100%, and detected recurrence 3.5 months earlier than CT. |
|  | 6 |  |  |  |  |  |  |  |  |  |  |
| Ryan BM; 2003 (33) | 123 (follow-up in n=94) | All stages | 66y | 60% | Mutation:  *KRAS2*  and β-actin for DNA integrity | serum, PCR | Pre-surgery and every 3 months until 3y | | ≤ 80 months | CEA | Pre-surgery *KRAS* mutation was not an independent predictor of recurrence. Post-operative *KRAS* mutation was associated with recurrence (HR 6.37). Plasma was positive 4 months prior to clinical diagnosis. Sensitivity and specificity for recurrence were 52.6% and 92%. CEA was elevated in 30% of the ctDNA positive cases that developed recurrence. |
|  | 20 |  |  |  |  |  |  |  |  |  |  |
| Schøler LV; 2017 (34) | 45 | All stages (with mutations identified in patient tumour tissue prior to plasma testing) | NA | NA | DNA copy number (CPP1) and mutations based on tumour tissue findings (including *KRAS* mutations) | Plasma, digital droplet PCR | Pre-surgery and follow-up at d8, d30 and every 3 months | | ≤ 60 months | CEA | ctDNA post-surgery was associated with recurrence which was detected 9.4 months before CT. CEA lead time was significantly less at 3.3 months. |
|  | 14 |  |  |  |  |  |  |  |  |  |  |
| Tie J; 2016, 2018 (35) (36) | 159;230 | Stage II CRC and Stage II-III rectal cancer (with mutations identified in patient tumour tissue prior to plasma testing) | 63y; 65y | 57%; 67% | Mutations based on tumour tissue findings (including *KRAS, APC, TP53* mutations) | Plasma, Safe-SeqS assay | Pre-treatment in rectal cases only, and at follow-up in all cases | | 24-27 months | CEA | Post-surgery presence of ctDNA was independently associated with recurrence-free survival (HR 6.0-14). CEA was not associated with recurrence-free survival in CRC stages II, but was for rectal cancer stage II-III (HR 5.1). ctDNA was more sensitive for recurrence in stage II CRC (85%) compared to CEA (41%). |
|  | 23; 34 |  |  |  |  |  |  |  |  |  |  |
| Wang JY; 2004 (37) | 104 | All stages | NA | 49% | Mutations:  *APC, p53, KRAS* | serum, PCR | Pre-surgery | | 20 months | CEA | ctDNA was significantly associated with recurrence incidence (*p*< 0.001). No statistical significance was observed between serum CEA levels and recurrence (*p*=0.247). |
|  | 31 |  |  |  |  |  |  |  |  |  |  |
| Xue H; 2017 (38) | 95 | All stages | 55y | 57% | Hypomethylation: *CBS* promoter | Plasma, PCR | Pre-surgery | | 37.5 months | No | Low methylation was associated with incidence of recurrence (incidence of 60.4% vs 26.2%) and was independently associated with recurrence-free survival (HR 1.62) |
|  | 43 |  |  |  |  |  |  |  |  |  |  |
| Young GP, 2016 (39) | 397 | All stages | 66y | 61% | Methylation: *BCAT1* and *IKZF1* | Plasma, PCR | Follow-up | | ≤ 41 months | CEA | Methylated ctDNA had a sensitivity for recurrence significantly higher than that for CEA (68% vs 32%) |
|  | 28 |  |  |  |  |  |  |  |  |  |  |
| Zhou J; 2016 (40) | 6 | Stage I-III | 64y (57-75y) | 67% | Mutations of 545 genes (including FBXW7, APC, AXIN2, TP53, KRAS) | Plasma, NGS | Pre-surgery, post-surgery and follow-up | | 32-47 months | CEA, CA 19-9 | ctDNA increased prior to recurrence (n=1), but CEA and CA 19-9 did not. |
|  | 1 |  |  |  |  |  |  |  |  |  |  |
| ***Survival*** | | | | | | | | | | | |
| **Author, year** | **Sample size** | **Cancer stage** | **Age (y, median or mean, range)** | **% Male** | **Biomarker** | **Method** | | **Sampling time** | **Follow-up time** | **Comparison to clinically used methods** | **Finding** |
|  | **No. deaths** |  |  |  |  |  |  |  |  |  |  |
| Bedin C; 2017 (41) | 114 | All stages | 67y | 49% | DNA fragments (*Alu83* and *Alu244*),  Methylation: *OSMR* and *SFRP* (relative to *MYOD*) | Plasma, PCR | | Pre-surgery / treatment | 52 months | No | Overall survival was approx. 1y less for cases with high *Alu83* and *Alu244*. Both were independently associated with survival (*Alu83* HR 2.71; *Alu244* HR 2.40). There was no association of methylation with survival. |
|  | 28 |  |  |  |  |  |  |  |  |  |  |
| Czeiger D; 2016 (42) | 37 | All stages | 68y | 45% | DNA levels | Serum, fluorescent assay | | Pre-surgery | > 12 months | CEA | Sensitivity for all-cause mortality was 92%, specificity was 72%. NPV=95%, PPV=91%. Sensitivity for disease-mortality was 100%, specificity was 66%. NPV=100%, PPV=44%. DNA levels were an independent predictor of overall survival (HR 3.53). |
|  | 6 |  |  |  |  |  |  |  |  |  |  |
| Garlan F; 2017 (43) | 82 (and 35 in validation cohort) | Stage IV (receiving 1^st^ or 2^nd^ line chemotherapy, with or without targeted therapy) | 68y | 60% | Mutations:  *KRAS, BRAF, TP53*  Methylation: *WIF1, NPY* | Plasma, digital droplet PCR | | Before 1^st^ (baseline), 2^nd^ and 3^rd^ cycle of chemotherapy | ≤ 40 months | CEA | Baseline ctDNA was independently associated with poorer overall survival (HR 3.36-12.65). High ctDNA had a shorter survival (6.8 months vs 33.4 months for those with no ctDNA). |
|  | NA |  |  |  |  |  |  |  |  |  |  |
| Guadalajara H; 2008 (27) | 73 | All stages | 68y | 64% | DNA level | Plasma, spectrophotometry (NanoDrop) | | Pre-surgery | 554 days (10-1407 days) | No | There was a trend, but no significant association between DNA level and overall survival. |
|  | 15 |  |  |  |  |  |  |  |  |  |  |
| Herbst A; 2017 (44) | 467 | Stage IV (treated with a combination therapy containing a fluoropyrimidine, oxaliplatin and bevacizumab) | NA | 64% | Methylation: *HPP1* (relative to *Alu*) | Plasma, PCR | | Prior to treatment and after 15-22 days. | ≤ 50 months | CEA | Cases with detectable methylated *HPP1* in either the 1^st^ or 2nd blood sample had a lower overall survival compared to no methylation (HR 1.86-2.13). High CEA gave similar results (HR 1.82-1.75). |
|  | 246 |  |  |  |  |  |  |  |  |  |  |
| Lecomte T; 2002 (29) | 37 | All stages (tumours with either *KRAS2* mutation or *p16* gene promoter methylation) | 69y | 50% | Methylation: *KRAS2* and *p16* (relative to unmethylated levels) | Plasma, PCR | | Pre-surgery | Mean 22 months | No | 2y overall survival was 48% for cases with ctDNA, compared to 100% without ctDNA. It was an independent predictor of survival (HR 10). |
|  | 19 |  |  |  |  |  |  |  |  |  |  |
| Lefebure B; 2010 (45) | 31 | Unresectable stage IV (treated with chemotherapy) | NA | NA | Mutation: *KRAS2*  Methylation: *RASSF2A* (relative to unmethylated levels) | Plasma, PCR | | Pre-surgery, post-surgery and follow-up | Median 11.5 months | No | At follow-up, 79% with ctDNA were deceased compared with 9% (p=0.001). |
|  | 12 |  |  |  |  |  |  |  |  |  |  |
| Li J; 2017(34) | 79 (follow-up n=35) | All stages (follow-up in stage III and IV) | 61y | 49% | DNA copy number variation | Plasma and serum, NGS | | Pre-surgery / treatment | Median 64 months (7-73 months) | No | Those with high copy numbers of 6 genomic regions had shorter survival (HR 5.33), with a median survival of 68.5 months vs 15.9 months) |
|  | 23 |  |  |  |  |  |  |  |  |  |  |
| Lin JK; 2014 (46) | 191 | All stages | 67y | 59% | DNA copy numbers (cyclophilin) and mutations:  *KRAS, APC, TP53, PIK3CA, BRAF* (genes of interest out of 74 genes) | Plasma, PCR | | Pre-surgery | 60 months (12-84 month) | CEA | 5y survival in cases with ctDNA mutation was lower than those without mutation (49% vs 77%, p=0.008). Survival was also lower with high DNA level vs low DNA level (43% vs 78%, p=0.001). High DNA level was independently associated with poorer survival (HR 2.61) as was CEA (HR 1.93). Mutation status of single genes was not associated with survival. |
|  | 62 |  |  |  |  |  |  |  |  |  |  |
| Liu Y; 2016 (30) | 165 | All stages | 67y | 55.2% | Methylation:  *SST, MAL, TAC1, SEPT9, EYA4, CRABp1, NELL* (normalised to β-actin) | Plasma, PCR | | Pre-surgery | Median 56 months | CEA | Cases with methylated *SST* or *MAL,* or high CEA were associated with lower cancer-specific survival. Only methylated *SST* was an independent predictor (HR 1.96). |
|  | 58 |  |  |  |  |  |  |  |  |  |  |
| Matthaios D; 2016 (47) | 155 | All stages | 70y | 57% | Methylation:  *APC, RASSF1A*) (relative to unmethylated levels) | Serum, PCR | | Pre-surgery | ≤ 100 months | CEA, CA 19-9 | Methylation of *APC* or *RASSF1A* were both associated with significantly shorter survival in early stage and stage IV CRC. Methylation was not significantly associated with CEA or CA 19-9. Methylated *APC* had highest independent prediction of survival in early stage (HR 7.88). Methylated *RASSF1A* had highest independent prediction of survival in stage IV (HR 5.76). |
|  | NA |  |  |  |  |  |  |  |  |  |  |
| El Messaoudi S; 2016 (48) | 97 | Stage IV | 67y | 60% | DNA concentration (using wild-type sequence) and mutations: *KRAS, BRAF* | Plasma, PCR | | At diagnosis | Median 36 months | CEA | CEA was not associated with overall survival. Total DNA level and BRAF mutation were independent predictors for poor survival (HR 1.73, HR 7.33) |
|  | NA |  |  |  |  |  |  |  |  |  |  |
| Philipp AB; 2012 (49) | 311 | All stages | NA | 55% | Methylation: *HLTF, HPP1* (normalised to *Alu*) | Serum, PCR | | Pre-treatment | ≤ 120 months | CEA | Shorter overall survival was found in stage I-III with methylated *HLTF* or *HPP1,* or CEA (p<0001). In stage IV all were independent predictors of survival (HR 1.6, HR 1.8, HR 1.7, respectively). |
|  | 190 |  |  |  |  |  |  |  |  |  |  |
| Philipp AB; 2014 (50) | 259 | All stages | NA | NA | Methylation: *HLTF, HPP1, NEUROG1* (normalised to *Alu*) | Serum, PCR | | Pre-treatment | ≤ 120 months | LDH | Shorter overall survival of stage IV was found with methylated *HLTF* (0.86 vs 1.6y), *HPP1* (1.0 vs 1.8y)*,* and high LDH (1.0 vs 1.8y). In stage I-III no biomarkers predicted survival |
|  | NA |  |  |  |  |  |  |  |  |  |  |
| Rasmussen SL; 2018 (51) | 193 | All stages | NA | 62% | Methylation: *ALX4, BNC1, HIC1, RARB, RASSF1A, SDC2, SEPT9, SRFP1, SRFP2, SPG20, TFP12, THBD, WIF1, APC, BMP3, BRCA1, CDKN2A, HLTF, MGMT, MLH1, NDRG4, NPTX2, NEUROG1, OSMR, PHACTR3, PPENK, SST, TAC1, VIM, WNT5A* | Plasma, PCR | | Pre-treatment | 60 months | CEA | Having >4 methylation markers decreased overall survival (p<0.001). Methylated *RARB* (HR 1.99) and *RASSF1A* (HR 3.35) were independent predictors of poor survival. |
|  | 74 |  |  |  |  |  |  |  |  |  |  |
| Schwarzenbach H; 2008 (52) | 55 | Stage IV | 63y | 77% | DNA level | Serum, spectrophotometry (NanoDrop) | | Pre-surgery and follow-up (n=14) | ≤ 120 months | No | High DNA level correlated with shorter survival (p=0.02) |
|  | 33 |  |  |  |  |  |  |  |  |  |  |
| Schøler LV; 2017 (34) | 21 | Stage I-III (with mutations identified in patient tumour tissue prior to plasma testing) | NA | NA | DNA copy number and mutations based on tumour tissue findings (including *KRAS*) | Plasma, digital droplet PCR | | Pre-surgery and follow-up at d8, d30 and every 3 months | ≤ 60 months | CEA | ctDNA was associated with a shorter overall survival (HR 6.7, p=0.01) |
|  | NA |  |  |  |  |  |  |  |  |  |  |
| Sefrioui D; 2015 (53) | 34 | Stage IV (undergoing chemotherapy) | 63y | 59% | DNA level and Mutations:  *KRAS* | Plasma, chip-based digital PCR | | Pre-treatment | ≤ 22 months | No | A high DNA level was associated with a low overall survival (4.8 months vs >22 months, p=0.024). Detectable ctDNA was also associated with a low overall survival (11.8 months vs >22 months, p=0.04). |
|  | NA |  |  |  |  |  |  |  |  |  |  |
| Shin SJ; 2017 (54) | 160 (follow-up n=17) | All stages | NA | NA | Mutation:  *KRAS* | Plasma, digital droplet PCR | | Pre-surgery and follow-up | ≤ 80 months |  | ctDNA correlated with shorter overall survival, p =0.003. |
|  | NA |  |  |  |  |  |  |  |  |  |  |
| Spindler KL; 2012, 2013, 2014, 2015 (55-58) | 49-229 | Stage IV (treated with cetuximab ± irinotecan, gemcitabine and capecitabine) | 62-66y | 55-77% | Mutations:  *KRAS* and *BRAF* and DNA copy number (cyclophilin) | Plasma, PCR | | Pre-treatment | 7-60 months | CEA, LDH | All studies showed that high levels of pre-treatment DNA level or *KRAS* mutations were associated with a poorer survival (DNA level assessed in 4 studies: HR 1.24, 1.5, 1.6, 1.7 and 1.8; *KRAS* mutation assessed in 3 studies HR 2.7, 3.14, 3.2; *BRAF* mutation assessed in 2 studies HR 4.3, 4.8). CEA was not associated with overall survival (assessed in one study). LDH was associated with survival (HR 1.31), but this was not an independent predictor. |
|  | NA in most studies.  90 in one study |  |  |  |  |  |  |  |  |  |  |
| Tie J; 2015 (59) | 53 | Stage IV (with mutations identified in patient tumour tissue prior to plasma testing) | 65y | 60% | Mutations based on tumour tissue findings (including *KRAS*) | Plasma, Safe-SeqS assay | | Pre-treatment, post-treatment | Approx 20 months | CEA | Fold change in ctDNA was not associated with overall survival. *Overall survival with CEA was not reported. |
|  | 20 |  |  |  |  |  |  |  |  |  |  |
| Xue H; 2017 (38) | 95 | All stages | 55y | 57% | Hypomethylation: *CBS* | Plasma, PCR | | Pre-surgery | 37.5 months | No | Low methylation was associated with poor 5y survival (21.4% vs 52.8%) and independently associated with overall survival (HR 1.49) |
|  | 37 |  |  |  |  |  |  |  |  |  |  |
| ***Disease-free survival (recurrence + death)*** | | | | | | | | | | | |
| **Author, year** | **Sample size** | **Cancer stage** | **Age (y)** | **% Male** | **Biomarker** | **Method** | | **Time of sample collection** | **Follow-up time** | **Comparison to clinically used methods** | **Finding** |
|  | **No. recurrence + death** |  |  |  |  |  |  |  |  |  |  |
| Bedin C; 2017 (41) | 114 | All stages | 67y | 49% | DNA fragments (*Alu83* and *Alu244*), and methylation:  *OSMR* and *SFRP* (relative to *MYOD*) | Plasma, PCR | | Pre-surgery / treatment | 52 months | No | There was no association of methylation with disease-free survival.  DNA fragments was independently associated with poor overall survival (*Alu83*: HR 2.71, p=0.015; *Alu244*: HR 2.40, p=0.023). |
|  | 6 |  |  |  |  |  |  |  |  |  |  |
| Czeiger D; 2016 (42) | 37 | All stages | 68y | 45% | DNA level | Serum, fluorescent assay | | Pre-surgery | 60 months | CEA | Sensitivity for disease-free survival was 100%, specificity was 68%. DNA levels were an independent predictor of overall survival (HR 6.03). CEA was not predictive of disease free survival (data not shown). NPV=100%, PPV=50% |
|  | NA |  |  |  |  |  |  |  |  |  |  |

ctDNA: circulating tumour DNA; NGS: next-generation sequencing; NA: not available; NPV: negative predictive value; PPV: positive predictive value.

**REFERENCES**

1. Eisenberger CF, Knoefel WT, Peiper M, Merkert P, Yekebas EF, Scheunemann P, et al. Squamous Cell Carcinoma of the Esophagus Can Be Detected by Microsatellite Analysis in Tumor and Serum. *Clinical Cancer Research* (2003) 9(11):4178-83.

2. Eisenberger CF, Stoecklein NH, Jazra S, Hosch SB, Peiper M, Scheunemann P, et al. The detection of oesophageal adenocarcinoma by serum microsatellite analysis. *European Journal of Surgical Oncology (EJSO)* (2006) 32(9):954-60. doi: 10.1016/j.ejso.2006.02.015.

3. Hsieh C-C, Hsu H-S, Chang S-C, Chen Y-J. Circulating Cell-Free DNA Levels Could Predict Oncological Outcomes of Patients Undergoing Esophagectomy for Esophageal Squamous Cell Carcinoma. *International Journal of Molecular Sciences* (2016) 17(12). doi: 10.3390/ijms17122131.

4. Ueda M, Iguchi T, Masuda T, Nakahara Y, Hirata H, Uchi R, et al. Somatic mutations in plasma cell-free DNA are diagnostic markers for esophageal squamous cell carcinoma recurrence. *Oncotarget* (2016) 7(38):62280-91. doi: 10.18632/oncotarget.11409.

5. Hoffmann A-C, Vallböhmer D, Prenzel K, Metzger R, Heitmann M, Neiss S, et al. Methylated DAPK and APC promoter DNA detection in peripheral blood is significantly associated with apparent residual tumor and outcome. *Journal of Cancer Research and Clinical Oncology* (2009) 135(9):1231-7. doi: 10.1007/s00432-009-0564-x.

6. Jin Z, Olaru A, Yang J, Sato F, Cheng Y, Kan T, et al. Hypermethylation of Tachykinin-1 Is a Potential Biomarker in Human Esophageal Cancer. *Clinical Cancer Research* (2007) 13(21):6293-300. doi: 10.1158/1078-0432.CCR-07-0818.

7. Ling ZQ, Zhao Q, Zhou SL, Mao WM. MSH2 promoter hypermethylation in circulating tumor DNA is a valuable predictor of disease-free survival for patients with esophageal squamous cell carcinoma. *European Journal of Surgical Oncology (EJSO)* (2012) 38(4):326-32. doi: 10.1016/j.ejso.2012.01.008.

8. Fang W-L, Lan Y-T, Huang K-H, Liu C-A, Hung Y-P, Lin C-H, et al. Clinical significance of circulating plasma DNA in gastric cancer. *International Journal of Cancer* (2016) 138(12):2974-83. doi: 10.1002/ijc.30018.

9. Hamakawa T, Kukita Y, Kurokawa Y, Miyazaki Y, Takahashi T, Yamasaki M, et al. Monitoring gastric cancer progression with circulating tumour DNA. *British Journal of Cancer* (2015) 112(2):352-6. doi: 10.1038/bjc.2014.609.

10. Lan Y-T, Chen M-H, Fang W-L, Hsieh C-C, Lin C-H, Jhang F-Y, et al. Clinical relevance of cell-free DNA in gastrointestinal tract malignancy. *Oncotarget* (2016) 8(2):3009-17. doi: 10.18632/oncotarget.13821.

11. Pu W-Y, Zhang R, Xiao L, Wu Y-Y, Gong W, Lv X-D, et al. Prediction of cancer progression in a group of 73 gastric cancer patients by circulating cell-free DNA. *BMC Cancer* (2016) 16. doi: 10.1186/s12885-016-2977-7.

12. Shoda K, Ichikawa D, Fujita Y, Masuda K, Hiramoto H, Hamada J, et al. Monitoring the HER2 copy number status in circulating tumor DNA by droplet digital PCR in patients with gastric cancer. *Gastric Cancer* (2017) 20(1):126-35. doi: 10.1007/s10120-016-0599-z.

13. Shoda K, Ichikawa D, Fujita Y, Masuda K, Hiramoto H, Hamada J, et al. Clinical utility of circulating cell-free Epstein–Barr virus DNA in patients with gastric cancer. *Oncotarget* (2017) 8(17):28796-804. doi: 10.18632/oncotarget.15675.

14. Balgkouranidou I, Matthaios D, Karayiannakis A, Bolanaki H, Michailidis P, Xenidis N, et al. Prognostic role of APC and RASSF1A promoter methylation status in cell free circulating DNA of operable gastric cancer patients. *Mutation Research/Fundamental and Molecular Mechanisms of Mutagenesis* (2015) 778:46-51. doi: 10.1016/j.mrfmmm.2015.05.002.

15. Balgkouranidou I, Karayiannakis A, Matthaios D, Bolanaki H, Tripsianis G, Tentes AA, et al. Assessment of SOX17 DNA methylation in cell free DNA from patients with operable gastric cancer. Association with prognostic variables and survival. *Clinical Chemistry and Laboratory Medicine* (2013) 51(7):1505-10. doi: 10.1515/cclm-2012-0320.

16. Kinugasa H, Nouso K, Tanaka T, Miyahara K, Morimoto Y, Dohi C, et al. Droplet digital PCR measurement of HER2 in patients with gastric cancer. *British Journal of Cancer* (2015) 112(10):1652-5. doi: 10.1038/bjc.2015.129.

17. Pimson C, Ekalaksananan T, Pientong C, Promthet S, Putthanachote N, Suwanrungruang K, et al. Aberrant methylation of PCDH10 and RASSF1A genes in blood samples for non-invasive diagnosis and prognostic assessment of gastric cancer. *PeerJ* (2016) 4. doi: 10.7717/peerj.2112.

18. Han J, Lv P, Yu J-L, Wu Y-C, Zhu X, Hong L-L, et al. Circulating Methylated MINT2 Promoter DNA Is a Potential Poor Prognostic Factor in Gastric Cancer. *Digestive Diseases and Sciences* (2014) 59(6):1160-8. doi: 10.1007/s10620-013-3007-0.

19. Ling Z-Q, Lv P, Lu X-X, Yu J-L, Han J, Ying L-S, et al. Circulating Methylated XAF1 DNA Indicates Poor Prognosis for Gastric Cancer. *PLoS ONE* (2013) 8(6). doi: 10.1371/journal.pone.0067195.

20. Yu J-L, Lv P, Han J, Zhu X, Hong L-L, Zhu W-Y, et al. Methylated TIMP-3 DNA in Body Fluids Is an Independent Prognostic Factor for Gastric Cancer. *Archives of Pathology & Laboratory Medicine* (2014) 138(11):1466-73. doi: 10.5858/arpa.2013-0285-OA.

21. Rawnaq T, Schwarzenbach H, Schurr PG, Freise K, Brandl S, Izbicki JR, et al. Monitoring of Loss of Heterozygosity in Serum Microsatellite DNA Among Patients with Gastrointestinal Stromal Tumors Indicates Tumor Recurrence. *Journal of Surgical Research* (2009) 169(1):31-5. doi: 10.1016/j.jss.2009.12.032.

22. Yoo C, Ryu MH, Na YS, Ryoo BY, Park SR, Kang YK. Analysis of serum protein biomarkers, circulating tumor DNA, and dovitinib activity in patients with tyrosine kinase inhibitor-refractory gastrointestinal stromal tumors. *Annals of Oncology* (2014) 25(11):2272-7. doi: 10.1093/annonc/mdu386.

23. Cassinotti E, Boni L, Segato S, Rausei S, Marzorati A, Rovera F, et al. Free circulating DNA as a biomarker of colorectal cancer. *International Journal of Surgery* (2013) 11:S54-S7. doi: 10.1016/S1743-9191(13)60017-5.

24. Diehl F, Schmidt K, Choti MA, Romans K, Goodman S, Li M, et al. Circulating mutant DNA to assess tumor dynamics. *Nature medicine* (2008) 14(9):985-90. doi: 10.1038/nm.1789.

25. Frattini M, Gallino G, Signoroni S, Balestra D, Battaglia L, Sozzi G, et al. Quantitative Analysis of Plasma DNA in Colorectal Cancer Patients. *Annals of the New York Academy of Sciences* (2006) 1075(1):185-90. doi: 10.1196/annals.1368.025.

26. Garrigou S, Perkins G, Garlan F, Normand C, Didelot A, Corre DL, et al. A Study of Hypermethylated Circulating Tumor DNA as a Universal Colorectal Cancer Biomarker. *Clinical Chemistry* (2016) 62(8):1129-39. doi: 10.1373/clinchem.2015.253609.

27. Guadalajara H, Domínguez-Berzosa C, García-Arranz M, Herreros MD, Pascual I, Sanz-Baro R, et al. The concentration of deoxyribonucleic acid in plasma from 73 patients with colorectal cancer and apparent clinical correlations. *Cancer Detection and Prevention* (2008) 32(1):39-44. doi: 10.1016/j.cdp.2008.01.002.

28. Kidess E, Heirich K, Wiggin M, Vysotskaia V, Visser BC, Marziali A, et al. Mutation profiling of tumor DNA from plasma and tumor tissue of colorectal cancer patients with a novel, high-sensitivity multiplexed mutation detection platform. *Oncotarget* (2014) 6(4):2549-61.

29. Lecomte T, Berger A, Zinzindohoué F, Micard S, Landi B, Blons H, et al. Detection of free‐circulating tumor‐associated DNA in plasma of colorectal cancer patients and its association with prognosis. *International Journal of Cancer* (2002) 100(5):542-8. doi: 10.1002/ijc.10526.

30. Liu Y, Chew MH, Tham CK, Tang CL, Ong SY, Zhao Y. Methylation of serum SST gene is an independent prognostic marker in colorectal cancer. *Am J Cancer Res* (2016) 6(9):2098-108.

31. Ng SB, Chua C, Ng M, Gan A, Poon PSY, Teo M, et al. Individualised multiplexed circulating tumour DNA assays for monitoring of tumour presence in patients after colorectal cancer surgery. *Scientific Reports* (2017) 7. doi: 10.1038/srep40737.

32. Reinert T, Schøler LV, Thomsen R, Tobiasen H, Vang S, Nordentoft I, et al. Analysis of circulating tumour DNA to monitor disease burden following colorectal cancer surgery. *Gut* (2016) 65(4):625-34. doi: 10.1136/gutjnl-2014-308859.

33. Ryan BM, Lefort F, McManus R, Daly J, Keeling PWN, Weir DG, et al. A prospective study of circulating mutant KRAS2 in the serum of patients with colorectal neoplasia: strong prognostic indicator in postoperative follow up. *Gut* (2003) 52(1):101-8.

34. Schøler LV, Reinert T, Ørntoft M-BW, Kassentoft CG, Árnadóttir SS, Vang S, et al. Clinical Implications of Monitoring Circulating Tumor DNA in Patients with Colorectal Cancer. *Clinical Cancer Research* (2017) 23(18):5437-45. doi: 10.1158/1078-0432.CCR-17-0510.

35. Tie J, Wang Y, Tomasetti C, Li L, Springer S, Kinde I, et al. Circulating tumor DNA analysis detects minimal residual disease and predicts recurrence in patients with stage II colon cancer. *Science translational medicine* (2016) 8(346):346ra92. doi: 10.1126/scitranslmed.aaf6219.

36. Tie J, Cohen JD, Wang Y, Li L, Christie M, Simons K, et al. Serial circulating tumour DNA analysis during multimodality treatment of locally advanced rectal cancer: a prospective biomarker study. *Gut* (2018):gutjnl-2017-315852. doi: 10.1136/gutjnl-2017-315852.

37. Wang J-Y, Hsieh J-S, Chang M-Y, Huang T-J, Chen F-M, Cheng T-L, et al. Molecular Detection of APC, K-ras, and p53 Mutations in the Serum of Colorectal Cancer Patients as Circulating Biomarkers. *World Journal of Surgery* (2004) 28(7):721-6. doi: 10.1007/s00268-004-7366-8.

38. Xue G, Lu C-J, Pan S-J, Zhang Y-L, Miao H, Shan S, et al. DNA hypomethylation of CBS promoter induced by folate deficiency is a potential noninvasive circulating biomarker for colorectal adenocarcinomas. *Oncotarget* (2017) 8(31):51387-401. doi: 10.18632/oncotarget.17988.

39. Young GP, Pedersen SK, Mansfield S, Murray DH, Baker RT, Rabbitt P, et al. A cross-sectional study comparing a blood test for methylated BCAT1 and IKZF1 tumor-derived DNA with CEA for detection of recurrent colorectal cancer. *Cancer Med* (2016) 5(10):2763-72. doi: 10.1002/cam4.868.

40. Zhou J, Chang L, Guan Y, Yang L, Xia X, Cui L, et al. Application of Circulating Tumor DNA as a Non-Invasive Tool for Monitoring the Progression of Colorectal Cancer. *PLoS ONE* (2016) 11(7). doi: 10.1371/journal.pone.0159708.

41. Bedin C, Enzo MV, Del Bianco P, Pucciarelli S, Nitti D, Agostini M. Diagnostic and prognostic role of cell-free DNA testing for colorectal cancer patients. *International Journal of Cancer* (2017) 140(8):1888-98. doi: 10.1002/ijc.30565.

42. Czeiger D, Shaked G, Sebbag G, Vakhrushev A, Flomboym A, Lior Y, et al. Elevated Cell-Free DNA Measured by a Simple Assay Is Associated With Increased Rate of Colorectal Cancer Relapse. *American Journal of Clinical Pathology* (2016) 145(6):852-7. doi: 10.1093/ajcp/aqw068.

43. Garlan F, Laurent-Puig P, Sefrioui D, Siauve N, Didelot A, Sarafan-Vasseur N, et al. Early Evaluation of Circulating Tumor DNA as Marker of Therapeutic Efficacy in Metastatic Colorectal Cancer Patients (PLACOL Study). *Clinical Cancer Research* (2017) 23(18):5416-25. doi: 10.1158/1078-0432.CCR-16-3155.

44. Herbst A, Vdovin N, Gacesa S, Philipp A, Nagel D, Holdt LM, et al. Methylated free-circulating HPP1 DNA is an early response marker in patients with metastatic colorectal cancer. *International Journal of Cancer* (2017) 140(9):2134-44. doi: 10.1002/ijc.30625.

45. Lefebure B, Charbonnier F, Fiore FD, Tuech JJ, Pessot FL, Michot F, et al. Prognostic Value of Circulating Mutant DNA in Unresectable Metastatic Colorectal Cancer. *Annals of Surgery* (2010) 251(2):275. doi: 10.1097/SLA.0b013e3181c35c87.

46. Lin J-K, Lin P-C, Lin C-H, Jiang J-K, Yang S-H, Liang W-Y, et al. Clinical Relevance of Alterations in Quantity and Quality of Plasma DNA in Colorectal Cancer Patients: Based on the Mutation Spectra Detected in Primary Tumors. *Annals of Surgical Oncology* (2014) 21(4):680-6. doi: 10.1245/s10434-014-3804-5.

47. Matthaios D, Balgkouranidou I, Karayiannakis A, Bolanaki H, Xenidis N, Amarantidis K, et al. Methylation status of the APC and RASSF1A promoter in cell-free circulating DNA and its prognostic role in patients with colorectal cancer. *Oncology Letters* (2016) 12(1):748-56. doi: 10.3892/ol.2016.4649.

48. El Messaoudi S, Mouliere F, Du Manoir S, Bascoul-Mollevi C, Gillet B, Nouaille M, et al. Circulating DNA as a Strong Multimarker Prognostic Tool for Metastatic Colorectal Cancer Patient Management Care. *Clin Cancer Res* (2016) 22(12):3067-77. doi: 10.1158/1078-0432.CCR-15-0297.

49. Philipp AB, Stieber P, Nagel D, Neumann J, Spelsberg F, Jung A, et al. Prognostic role of methylated free circulating DNA in colorectal cancer. *International Journal of Cancer* (2012) 131(10):2308-19. doi: 10.1002/ijc.27505.

50. Philipp AB, Nagel D, Stieber P, Lamerz R, Thalhammer I, Herbst A, et al. Circulating cell-free methylated DNA and lactate dehydrogenase release in colorectal cancer. *BMC Cancer* (2014) 14:245. doi: 10.1186/1471-2407-14-245.

51. Rasmussen SL, Krarup HB, Sunesen KG, Johansen MB, Stender MT, Pedersen IS, et al. The prognostic efficacy of cell-free DNA hypermethylation in colorectal cancer. *Oncotarget* (2018) 9(6):7010-22. doi: 10.18632/oncotarget.24097.

52. Schwarzenbach H, Stoehlmacher J, Pantel K, Goekkurt E. Detection and Monitoring of Cell‐Free DNA in Blood of Patients with Colorectal Cancer. *Annals of the New York Academy of Sciences* (2008) 1137(1):190-6. doi: 10.1196/annals.1448.025.

53. Sefrioui D, Sarafan-Vasseur N, Beaussire L, Baretti M, Gangloff A, Blanchard F, et al. Clinical value of chip-based digital-PCR platform for the detection of circulating DNA in metastatic colorectal cancer. *Digestive and Liver Disease* (2015) 47(10):884-90. doi: 10.1016/j.dld.2015.05.023.

54. Shin S-J, Chun S-M, Kim T-I, Kim YJ, Choi H-J, Jang SJ, et al. Feasibility of multiplexed gene mutation detection in plasma samples of colorectal cancer patients by mass spectrometric genotyping. *PLoS ONE* (2017) 12(5):e0176340.

55. Spindler K-LG, Pallisgaard N, Vogelius I, Jakobsen A. Quantitative Cell-Free DNA, KRAS, and BRAF Mutations in Plasma from Patients with Metastatic Colorectal Cancer during Treatment with Cetuximab and Irinotecan. *Clinical Cancer Research* (2012) 18(4):1177-85. doi: 10.1158/1078-0432.CCR-11-0564.

56. Spindler KG, Appelt AL, Pallisgaard N, Andersen RF, Jakobsen A. KRAS-mutated plasma DNA as predictor of outcome from irinotecan monotherapy in metastatic colorectal cancer. *British Journal of Cancer* (2013) 109(12):3067-72. doi: 10.1038/bjc.2013.633.

57. Spindler K-LG, Pallisgaard N, Andersen RF, Ploen J, Jakobsen A. Gemcitabine and Capecitabine for Heavily Pre-treated Metastatic Colorectal Cancer Patients – A Phase II and Translational Research Study. *Anticancer Research* (2014) 34(2):845-50.

58. Spindler KLG, Pallisgaard N, Andersen RF, Brandslund I, Jakobsen A. Circulating Free DNA as Biomarker and Source for Mutation Detection in Metastatic Colorectal Cancer. *PLoS ONE* (2015) 10(4). doi: 10.1371/journal.pone.0108247.

59. Tie J, Kinde I, Wang Y, Wong HL, Roebert J, Christie M, et al. Circulating tumor DNA as an early marker of therapeutic response in patients with metastatic colorectal cancer. *Annals of Oncology* (2015) 26(8):1715-22. doi: 10.1093/annonc/mdv177.
